# Metal‐Free Boron/Phosphorus Co‐Doped Nanoporous Carbon for Highly Efficient Benzyl Alcohol Oxidation

**DOI:** 10.1002/advs.202200518

**Published:** 2022-04-11

**Authors:** Juan Meng, Zhihan Tong, Haixin Sun, Yongzhuang Liu, Suqing Zeng, Jianing Xu, Qinqin Xia, Qingjiang Pan, Shuo Dou, Haipeng Yu

**Affiliations:** ^1^ Key Laboratory of Bio‐Based Material Science and Technology of Ministry of Education Northeast Forestry University Harbin 150040 China; ^2^ Key Laboratory of Functional Inorganic Material Chemistry School of Chemistry and Materials Science Heilongjiang University Harbin 150080 China

**Keywords:** alcohol oxidation, boron, carbon, cellulose, codoping, metal‐free catalysts

## Abstract

An in‐depth understanding of the electronic structures of catalytically active centers and their surrounding vicinity is key to clarifying the structure–activity relationship, and thus enabling the design and development of novel metal‐free carbon‐based materials with desired catalytic performance. In this study, boron atoms are introduced into phosphorus‐doped nanoporous carbon via an efficient strategy, so that the resulting material delivers better catalytic performance. The doped B atoms alter the electronic structures of active sites and cause the adjacent C atoms to act as additional active sites that catalyze the reaction. The B/P co‐doped nanoporous carbon shows remarkable catalytic performance for benzyl alcohol oxidation, achieving high yield (over 91% within 2 h) and selectivity (95%), as well as low activation energy (32.2 kJ mol^−1^). Moreover, both the conversion and selectivity remain above 90% after five reaction cycles. Density functional theory calculations indicate that the introduction of B to P‐doped nanoporous carbon significantly increases the electron density at the Fermi level and that the oxidation of benzyl alcohol occurs via a different reaction pathway with a very low energy barrier. These findings provide important insights into the relationship between catalytic performance and electronic structure for the design of dual‐doped metal‐free carbon catalysts.

## Graphical abstract

Modulating the electronic state of the active sites and their surrounding vicinity in metal‐free carbon materials is a promising strategy for receiving highly efficient catalysts. This study realizes the facile preparation of boron/phosphorus codoped nanoporous carbon with remarkable catalytic performance, which enables the oxidation of benzyl alcohol occurred via a different reaction pathway with a very low energy barrier.

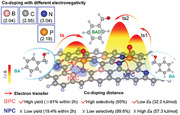

## 1 Introduction

The electronic states of the catalytically active sites play a vital role in determining the intrinsic activity of a catalyst.^[^ ^1^, ^2^, ^3^ ^]^ Favorable electronic characteristics, including electron density,^[^ ^4^ ^]^ value‐band structure,^[^ ^5^ ^]^ and spin state,^[^ ^6^ ^]^ can balance the reaction enthalpies of various species (e.g., reactants, intermediates, transition states, and products) along the reaction coordinates, facilitating suitable reactant adsorption, fast catalytic response, and easy desorption of products. Numerous strategies have been utilized to optimize the electronic structure of the catalytically active centers. The introduction of suitable foreign atoms (i.e., dopants) near the active sites of a catalyst can alter its behavior toward higher activity and selectivity.^[^ ^7^, ^8^ ^]^ For example, substitution of the coordinated nitrogen atom in the M—N*_x_* component (M = iron, nickel, cobalt, etc.) with oxygen, sulfur, or phosphorus has been widely adopted to prepare single‐atom catalysts.^[^ ^9^, ^10^, ^11^, ^12^, ^13^, ^14^ ^]^ Codoping with multiple heteroatoms to alter the electronic structure of the active sites is also one of the most effective ways to improve the catalytic activity of metal‐free materials.^[^ ^15^, ^16^, ^17^ ^]^ These findings provide important insights into the relationship between catalytic activity and electronic structures. However, the synergistic modulation of codopants and the corresponding active sites involved in the catalytic reaction remains challenging, which impacts the rational design of high‐performance metal‐free catalysts in this burgeoning research area.^[^ ^18^, ^19^ ^]^

Carbon‐based materials with a low price, stable structure, rich defect sites, tunable electronic structure, etc., have attracted much attention as metal‐free catalysts for many chemical reactions, such as organic synthesis, as well as electrochemical and photocatalytic reactions.^[^ ^20^, ^21^, ^22^ ^]^ The introduction of secondary heteroatoms with different electronegativities into carbon materials has been shown to produce multiple active centers with extraordinary catalytic functions. In our previous study, P‐doped nanoporous carbon (PC) with a high dopant content was fabricated as an efficient carbon‐based catalyst.^[^ ^23^ ^]^ A facile synthesis was developed by dissolving cellulose in phosphoric acid and directly carbonizing the resultant cellulose/phosphoric supramolecular collosol to successfully produce PC with a high loading and good dispersion of the P dopant. Although this synthesis facilitated a high conversion of benzyl alcohol (BA) to benzaldehyde (BAD) with the C_3_PO moiety as the optimal active center, the strategy of introducing secondary heteroatoms into PC to leverage the synergistic effect of two dopants is still appealing and encouraged.^[^ ^24^, ^25^, ^26^, ^27^ ^]^ The optimization of the catalytic active centers using secondary heteroatoms, according to different electronic states, remains attractive.

**Scheme 1:**
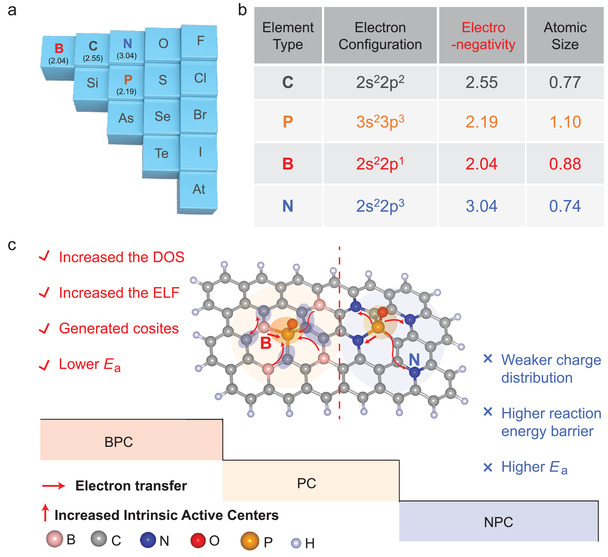
Heteroatom codoping for BA oxidation activity. a) Position of the heteroatoms of interest in the periodic table. b) Electron arrangement, electronegativity, and atomic size of the heteroatoms. c) Comparison of the electronic states, active sites, and activation energies between BPC and NPC.

Determining the type of secondary heteroatom that can be the most suitable partner of P for the as‐obtained PC is of interest. On the periodic table, B and N are neighbors of C and are located in the row above P but have distinct electronegativities (**Scheme** **1**). To investigate the relationship between the electronic structure of an active center and its activity when a secondary heteroatom is introduced, we prepared B/P codoped nanoporous carbon (BPC) and N/P codoped nanoporous carbon (NPC). Surprisingly, significant differences in behavior were observed when these materials were used in the catalytic oxidation of BA to BAD. A much higher yield and selectivity to BAD were observed with BPC than with NPC. The most significant differences between these materials were ascribed to the manner in which the introduction of secondary heteroatoms changed the electronic structures of the active centers in PC, inducing an alternate reaction pathway and consequently lowering the kinetic energy barrier of the reaction. A series of theoretical calculations and experimental surveys were then conducted to obtain an in depth understanding of the internal mechanisms and regulations.

## 2 Results and Discussions

### 2.1 Density Functional Theory (DFT) Calculations for Electronic Configurations

**Figure 1:**
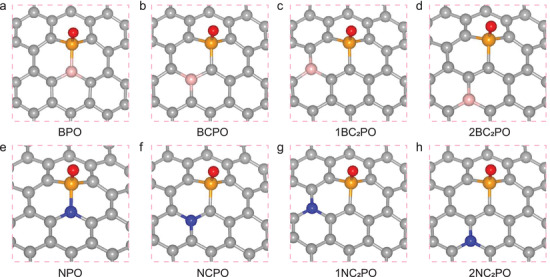
Local atomic configurations of BPC and NPC: a) BPO, b) BCPO, c) 1BC_2_PO, and d) 2BC_2_PO; e) NPO, f) NCPO, g) 1NC_2_PO, and h) 2NC_2_PO. The gray, red, yellow, pink, and blue balls, denote C, O, P, B, and N atoms, respectively.

The combination of different heteroatoms may cause structural distortions and changes in the charge density of carbon materials, leading to different local electronic effects on the active centers. It has been shown that the high catalytic activity of PC for BA oxidation originates from the C_3_PO moiety investigated in our previous study (see Figure S1, Supporting Information).^[^ ^23^ ^]^ However, incorporating a secondary heteroatom that differs significantly in electronegativity would lead to a withdrawing or donating effect on the electronic density, affecting the electronic structures of the active centers in the carbon material. To explore the influence of weakly electronegative B (2.04) or strongly electronegative N (3.04) on the catalytic activity of the parent PC material, different models with B/N and P atoms incorporated into the carbon layer and their electronic properties were first studied using the DFT (detailed under the Methods Section in the Supporting Information). The most stable configurations of the codoped heteroatoms for BPC are as follows: i) BPO with covalently bonded B−P, ii) BCPO with one C atom located between the B and P atoms, iii) 1BC_2_PO, and iv) 2BC_2_PO with two C atoms located between the B and P atoms (see **Figure** **1**). Accordingly, the configurations of the active centers of NPC are similar to those of BPC, i.e., NPO, NCPO, 1NC_2_PO, and 2NC_2_PO (see Figures S2 and S3, Supporting Information). The P═O active site protrudes from the carbon plane because of the large radius of P as well as the differences that exist between the valence electron configurations P (3s^2^3p^3^) and C (2s^2^2p^2^). The different electronegativities of B/N atoms can affect the electronic structure of the P═O active site and surrounding C atoms.^[^ ^4^, ^28^ ^]^ Moreover, the longer the distance between the secondary heteroatom and P, the weaker effect of the heteroatom on the electronic structure of P═O active site. Thus, the electronic structures of the four abovementioned configurations were prioritized.

**Figure 2:**
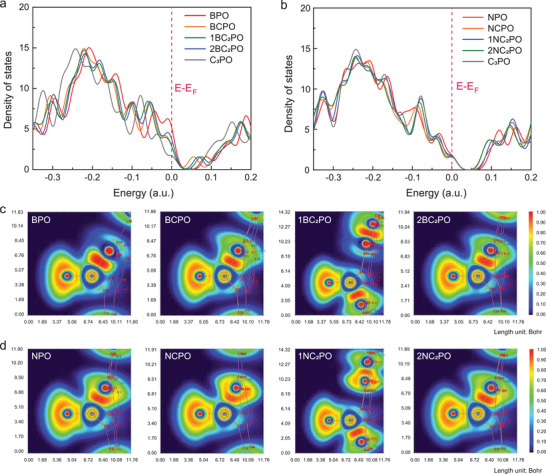
TDOS plots of the a) BPC and b) NPC configurations. ELFs of c) BPC and d) NPC corresponding to different configurations. The red color in this map reveals the high electron localization nature in the bonding regions.

The electronic properties in terms of the total density of states (TDOS) and projected density of states (PDOS) for the different configurations in BPC and NPC are illustrated in **Figure** **2**; and Figures S4 and S5 (Supporting Information). The electron centers of BPC shift toward the Fermi level and the electron density near the Fermi level increases significantly (see Figure 2a), indicating that B doping results in a more active electronic state of BPC than that of NPC. The direct B—P covalent bond at these positions contributes the most to the Fermi level, and the DOS changes significantly. The B dopant also changes the bandgap of the electronic structure near the Fermi level. The smaller the bandgaps in the BPC configurations, the stronger ability of the active sites to control the H dissociation of BA.^[^ ^29^, ^30^ ^]^ The bandgap of BCPO is the smallest among the various BPC configurations, indicating that its surface structure is the most suited for controlling the H dissociation of BA. These results indicate that the B dopant in the graphitic P=O active center can promote electron transfer and improve the activation of the reactants, which ultimately increase the catalytic activity of the system.^[^ ^31^, ^32^ ^]^ In contrast, no significant changes are observed at the Fermi level of NPC (see Figure 2b; and Figure S5, Supporting Information) and PC (see Figure S6, Supporting Information). The increased bandgap of NPO and the lower distribution of the valence band near the Fermi level may have a detrimental effect on the electron transfer rate of NPC.

We further compared the topologies of the Mulliken charges and electron localization functions (ELFs) to reflect the effects of the heteroatom codopants on the space charge distributions. The heteroatom dopant clearly influences the O, P, and C atoms in the catalyst structures of BPC (see Table S1, Supporting Information) and NPC (see Table S2, Supporting Information). Interestingly, two heteroatoms with different electronegativities reduce the positive charge of P in the graphitic carbon structure. Compared with the distance between O and P in PC, the shorter distance between B and P in BPC increases the negative charge on O, which acts as the main activation site for BA oxidation over BPC, with the exception of 2BC_2_PO (−0.556 e). This is because the codoped heteroatoms are not on the same aromatic ring structure, which significantly increases the conjugation effect and electronic delocalization of the graphitic carbon.^[^ ^33^, ^34^ ^]^ The increased negative charge of the C atoms around B and the reduced positive charge of P in the graphitic carbon of BPC may enhance *π*–*π* interactions between BA and BPC to facilitate the dissociation of H during BA oxidation. This also contributes to the formation of a more stable transition state (ts) and an optimal reaction pathway.^[^ ^35^ ^]^

Topographic analysis of the ELFs was performed in a 2D graph with the plane defined by the C, P, O, and B/N atoms, as shown in Figure 2c,d; and Figures S7–S9 (Supporting Information). The introduction of secondary heteroatoms has different effects on the ELFs of the bonding atoms in the local active sites. The B dopant significantly increases the localization strength of electrons accompanied by a deepened orange color, indicating a stronger ability to share electrons between the B—P and B—C bonds, especially when B and P are directly covalently bonded in BPO. The electronic localization in these active sites would be weaker than that in BPO but higher than that in C_3_PO, as the electronegativity of B is lower than that of C and P. The B dopant significantly enhances the local electron density of BPC, which is conducive to the movement of valence electrons in the active sites and increases the rate of the catalytic oxidation reaction.^[^ ^36^, ^37^ ^]^ Owing to the electron‐withdrawing ability of the electronegative N atom, the connected C atoms donate electrons in NPC, enhancing the positive charges of the C atoms and decreasing the localization strength of the valence electrons in the P—N and N—C bonds. This may not be beneficial for generating additional active sites with a certain negative effect on redox reactions.^[^ ^38^, ^39^ ^]^

### 2.2 Structural and Spectroscopic Characterizations

**Figure 3:**
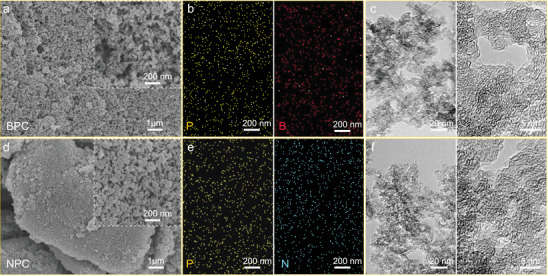
SEM images and SEM−EDS element maps of a,b) BPC and d,e) NPC. High‐resolution TEM images of c) BPC and f) NPC.

The supramolecular collosol doping strategy leads each cellulose molecular chain to be completely separated by the presence of phosphorus acid and fully combined with the precursors of the secondary heteroatoms.^[^ ^23^ ^]^ A closer connection with the heteroatoms before carbonization would be beneficial for realizing codoped carbon materials with well‐dispersed and highly loaded heteroatoms. The morphologies of BPC and NPC were determined by scanning electron microscopy–energy dispersive X‐ray spectroscopy (SEM–EDS) and transmission electron microscopy (TEM). Both codoped nanoporous carbon materials had similar morphologies. The SEM–EDS mapping images confirmed the homogeneous codoping and dense distribution of P/B atoms in BPC (see **Figure** **3**a,b) and P/N atoms in NPC (Figure 3d,e). Graphitization and amorphous carbon regions could be observed in the high‐resolution TEM images in Figure 3c,f. These results are consistent with the characteristics of PC (see Figure S10, Supporting Information). Notably, the introduction of secondary heteroatoms has little influence on the basic physical structure of the carbon nanoparticles (Figure S11, Supporting Information).^[^ ^40^ ^]^ Thus, the inherited structure (with a large surface area and pore volume) provides capacious channels for the transportation and diffusion of reactants and for promoting the reaction kinetics (see Table S3 and Figure S12, Supporting Information).^[^ ^41^ ^]^ BPC exhibits a higher specific surface area and more defects than NPC does, which is related to the nature of the second heteroatomic precursor. When compared with the B precursor (viz., sodium tetraborate hydrate), dicyandiamide served as a doping precursor with a higher N content and an additional carbon precursor during carbonization, together with the cellulose supramolecular collosol. The additional carbon may permeate into the carbon skeleton, resulting in a reduced specific surface area of NPC.^[^ ^42^, ^43^ ^]^ All of these phenomena would aid in improving the mass transfer process and reaction kinetics of BA oxidation.^[^ ^44^, ^45^ ^]^

**Figure 4:**
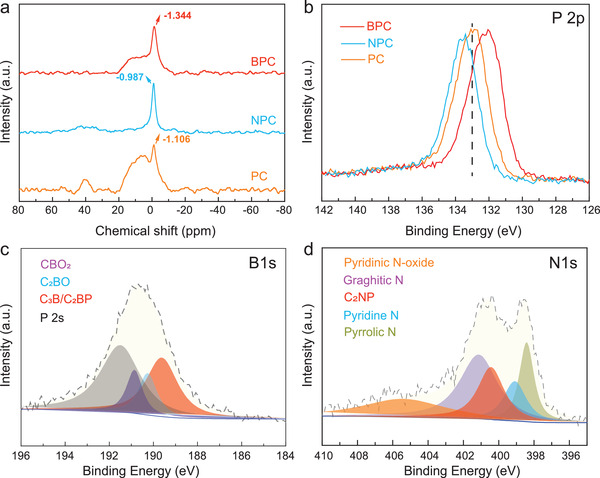
a) Solid‐state ^31^P NMR spectra; b) XPS survey of P 2p; c,d) High‐resolution XPS peaks of c) B 1s of BPC and d) N 1s of NPC.

Solid‐state ^31^P NMR (nuclear magnetic resonance) analysis was performed to investigate the influence of B or N on the chemical structure of BPC and NPC, respectively (see **Figure** **4**a). The NMR signal for BPC at −1.344 ppm shows an obvious negative shift when compared with that of PC (−1.106 ppm), with a positive shift observed for NPC (−0.987 ppm). Owing to the relationship between the empty and full‐orbit energy spacing, this difference likely originates from the electronic structure changes of P caused by the doped secondary heteroatom.^[^ ^46^, ^47^ ^]^ The electronegativity of B is lower than that of P, causing the signal to shift to a lower band, whereas the stronger electronegativity of N shifts the signal to a higher band. This position difference is attributed to the formation of triphenylphosphine oxide (O═PPh_3_, expected value of +28 ppm) and triphenylphosphine (PPh_3_, expected value of −5 ppm),^[^ ^48^, ^49^ ^]^ indicating that the carbonization process induced the reduction of the phosphoric acid groups to graphite phosphorus and other organic phosphonates with high oxidation states and C—P bonding.

X‐ray photoelectron spectroscopy (XPS) measurements were performed to study the chemical state and bonding structure of the heteroatoms to the carbon substrate (see Figure 4b–d; and Table S4, Supporting Information). The B dopant shifts the P 2p peak in BPC to a lower binding energy (132.2 eV) when compared with that of PC (132.9 eV), whose peak can be deconvoluted into the spectral contributions from C_3_PO, C_2_PO_2_/CPO_3_, and COPO_3_ (see Figures S13–S15, Supporting Information). The fitting of the B 1s peak is complicated because of the overlap of B 1s and P 2s, which can be deconvoluted into three peaks, as shown in Figure 4c, with the following assignments: CBO_2_ (190.9 eV), C_2_BO (190.3 eV), and C_3_B/C_2_BP (189.6 eV).^[^ ^50^, ^51^ ^]^ The introduction of B leads to an increase in the electron density of the outer layer of P, which decreases the electron binding energy of the nucleus and causes a shift of the P 2p peak to a lower binding energy (note that the opposite effect is observed in the presence of N). Furthermore, Fourier transform infrared (FTIR) spectroscopy reveals that the characteristic functional groups of BPC are similar to those of PC (see Figure S16, Supporting Information). The characteristic bands of P═O, P—O/C—O bends, and P—C vibrations are observed at 1145, 1020–1100, and 600–650 cm^−1^, respectively, while the characteristic bonds of the C═C stretching vibration of the aromatic groups appear at 1570 and 885 cm^−1^. The P═O band shows a blue shift owing to the presence of C—B in BPC.^[^ ^23^, ^31^, ^52^ ^]^ These results agree with the aforementioned electronic structures obtained from the DFT calculations.

### 2.3 Catalytic Performance of BPC

**Figure 5:**
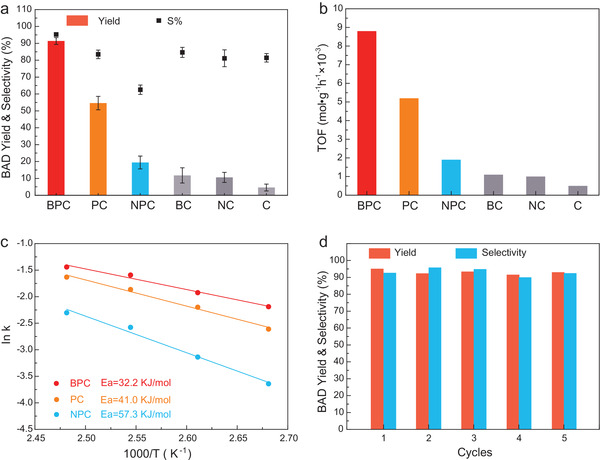
Catalytic performance of the catalysts in the oxidation of BA to BAD. Comparison of the a) BAD yield and selectivity, b) TOFs, and c) Arrhenius plots. d) Cycling evaluations for assessing the reusability of BPC.

The oxidation of BA to BAD is selected to investigate the catalytic performance of BPC, with NPC and PC as references. Excellent catalytic activity is achieved with BPC because of the favorable electronic state of the active sites. The conversion of BA reaches 91.4% at 130 °C within 2 h, with selectivity to BAD of more than 95% (see **Figure** **5**a), both of which are higher than those achieved by PC. In contrast, the conversion of BA over NPC under the same reaction conditions is below 30% with selectivity to BAD of 69.6%. This is related to the high N content, which results in the formation of an NPO structure in the NPC catalyst, thereby increasing the dissociation of the C—H bond of BA and significantly decreasing the catalytic performance.^[^ ^53^, ^54^, ^55^ ^]^ The intrinsic activities of the catalysts were further investigated, as shown in Figure 5b. The turnover frequency (TOF) of BPC is 8.8 × 10^−3^ mol g^−1^ h^−1^ (4.6 and 1.7 times higher than that of NPC and PC, respectively). This value is remarkably higher than those of most currently reported metal‐free and metal‐based single‐atom catalysts (see Table S5, Supporting Information). The apparent activation energy *E*_a_ was calculated to be 32.2, 41.0, and 57.3 kJ mol^−1^ for BPC, PC, and NPC, respectively (see Figure 5c), indicating that the highest catalytic activity is achieved when B is doped. The stability of the BPC catalyst is also excellent, with the conversion and selectivity exceeding 90% after five successive cycles (see Figure 5d). Taken together, the introduction of B to PC greatly increases the yield and selectivity to BAD, whereas the incorporation of N shows an adverse effect. Moreover, single‐doped materials (i.e., BC and NC), excluding P‐doping, do not display significant catalytic activity in the oxidation of BA (see Table S6, Supporting Information).

All the BPCs significantly improve the yield and selectivity to BAD when compared with PC. Combined with the XPS results in Table S4 (Supporting Information), increasing the amount of the B precursor increases the amount of B atoms loaded in the BPC catalysts. However, the P content shows a trend of first increasing and then decreasing with the continued addition of the B precursor, which is related to the solubility of the supramolecular collosol. The BPC collosol becomes milky and cloudy during the dissolution process, resulting in an adverse effect on P doping and reduces catalytic activity during the oxidation of BA (see Table S6, Supporting Information). Therefore, in our study, the BPC catalyst is optimized as a synergistic and highly active codoped carbon‐based catalyst. The results indicate that doped secondary heteroatoms with different electronegativities in PC can cause the catalytic performance of PC to change significantly. Therefore, our BPC is an excellent example for the design of advanced metal‐free nanoporous carbon catalysts.

### 2.4 Catalytic Mechanism

**Figure 6:**
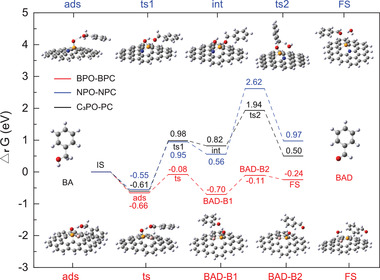
DFT studies on the catalytic reaction mechanism of the oxidation of BA: BPO configuration of BPC, NPO configuration of NPC, and C_3_PO configuration of PC. Marked energies for the target product BAD adsorbed on the intermediate structure of BPO (**BAD‐B1**), BAD desorption and the intermediate state of BPO (**BAD‐B2**), adsorption (**ads**), intermediate (**int**) formation, and transition state (**ts**) formation during the stepwise oxidation of the models. The C, O, P, B, N, and H atoms are indicated in gray, red, yellow, pink, blue, and white, respectively.

The mechanism of the catalytic oxidation of BA to BAD over different BPC configurations was elucidated by DFT calculations. The reaction energy profiles and structures of all the species along the reaction coordinates are shown in **Figure** **6**. During the oxidation of BA, the hydroxyl H (—OH) of BA is first captured by the P═O part of each model (BC_2_PO in BPO and C_3_PO for the other materials). This is a spontaneous process with a reaction free energy ranging from −0.55 to −0.66 eV. The doped secondary heteroatoms have different effects on the elimination of the second H atom. The B atom (with its weak electronegativity) activates the surrounding C atoms, making them function as additional catalytic centers owing to the enhanced negative charge and ELF of the B—C bond. In this case, the newly created active C centers are responsible for removing the H of the C—H bond in BA. Notably, BPC simultaneously activates the H atom of the O—H and C—H bonds, and the oxidation reaction of BA forms a unique **ts** with a very low energy barrier (see Figure 6). This is different from the trend observed for the C_3_PO configuration of PC, where the second H is eliminated by the same oxo‐atom of P═O (see Figure S17, Supporting Information). This results in the formation of two successive transition states of higher energy.

The position of the B dopant has a considerable effect on the reaction barrier. As shown in the catalytic reactions triggered by the four configurations of BPC in Figure S18 (Supporting Information), BPO exhibits the lowest adsorption energy (−0.66 eV), where a covalent bond between B and P is formed. The activation energy of the **ts** is only 0.74 eV to produce BAD‐B1, leading to the target product BAD. Thereafter, the separation of BAD and the catalyst via dehydration is completed at the cost of low less energy, and the P(III) species are oxidized by oxygen molecules and regenerated back to O═P(V) to close the catalytic cycle. All four BPC configurations have similar reaction pathways. In addition, they are energetically favored over the C_3_PO configuration of PC. The longer the distance is between B and P in each configuration, the less significant is the electronic collaboration between them and the closer the decisive energy barrier (1.48 vs 1.59 eV) is between 2BC_2_PO in BPC and C_3_PO in PC. Owing to the shorter distance between B and P, the charge effect between the active sites becomes more significant.

As seen in Figure 6; and Figure S19 (Supporting Information), the doping of N atoms into PC and their position in NPC does not produce significant changes in the reaction pathways, particularly in the initial two steps. In particular, for NPO, where P—N is covalently bonded, the energy barrier of the rate‐determining step (**ts2**) significantly increases (2.06 eV). This is caused by the manner in which **ts2** is greatly destabilized (Δ*_r_G* = 2.62 eV) and by the stabilization of its preceding intermediate (0.56 eV). Consequently, N doping increases the energy barrier for activating the C—H bond with respect to C_3_PO‐PC. Thus, the entire reaction triggered by NPC requires more external energy.

Briefly, PC and NPC show almost the same reaction pathway, in which two H atoms of BA (one from O—H and the other from C—H) are eliminated in sequence via two energy‐demanding transition states. In contrast, BPC adopted a different reaction pathway that not only allows for a one‐step dihydrogen elimination of BA, but also greatly lowers the energy barrier striding over the transition state. As a result, the incorporation of B heteroatoms into PC improves the catalytic performance of the BPC catalyst.

### 2.5 Extended Application Toward Catalytic Oxidation of Various Alcohols

**Figure 7:**
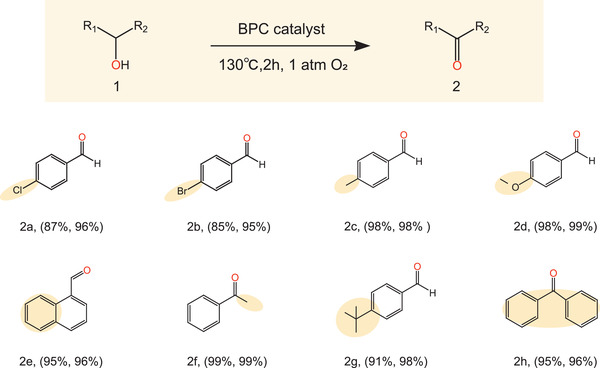
Catalytic oxidation of different alcohols using the BPC catalyst.

These excellent results encouraged us to explore the range of the BPC catalyst in terms of oxidizing other types of BAs, for example, secondary alcohols. As shown in **Figure** **7**, the BPC catalyst effectively catalyzes the conversion of both primary and secondary BAs to their corresponding aldehydes and ketones. All the chemicals show excellent selectivities (over 95%) and yields (85–99%). None of the BAs produce excess oxidation products during the catalytic process, indicating that BPC shows high selectivity for the production of aldehydes/ketones. The similar catalytic efficiencies observed for primary and secondary alcohols demonstrate the substrate universality of the BPC catalyst.

## 3 Conclusion

Our current study successfully demonstrated that B is the optimal partner of P as a heteroatom codopant in nanoporous carbon. A combination of theoretical calculations and experimental results revealed that BPC delivered superior catalytic performance for alcohol oxidation when compared with NPC or PC. Surprisingly, BPC and NPC showed an inverse catalytic effect when compared with that of PC. This can be attributed to the different electronegativities of B and N, the rearrangement of electron density at the Fermi level, the charge of the active sites, and changes in the bandgap. Consequently, BPC adopted an entirely different but more kinetically and thermodynamically favored reaction pathway in which the BPO configuration played a vital role. In brief, this study provides a new idea for the design of highly‐active metal‐free codoped carbon materials that are anticipated to replace metal‐based catalysts, and regulation of the electronic state is shown to be an effective strategy in the same manner.

## 4 Experimental Section

### Materials

*α*‐Cellulose (25 µm), phosphoric acid (85 wt%), sodium tetraborate decahydrate, dicyandiamide, zinc chloride, alcohols, and aldehyde were purchased from Aladdin Reagent Co., Ltd. (Shanghai, China) and Macklin Biochemical Co., Ltd. (Shanghai, China). All chemicals were used as received without further purification.

### Preparation of the Doped Nanoporous Carbon Catalysts

BPC and NPC catalysts: *α*‐Cellulose (3 g) and phosphoric acid (17 g) were added to a round‐bottom flask with vigorous stirring at room temperature until the cellulose was entirely dissolved (within 2 h) to generate a cellulose/phosphoric supramolecular collosol.^[^ ^23^ ^]^ Then, sodium tetraborate decahydrate (1.5 g) or dicyandiamide (1 g) was added to the above supramolecular collosol with continuous stirring until completely dissolved. The codoped supramolecular collosol precursor was transferred to a graphite crucible and carbonized under Ar atmosphere in a tube furnace at 600 °C for 2 h. The obtained carbon samples were further treated with 50 mL of 1 m HCl at 120 °C for 6 h to eliminate residual chemicals, washed with distilled water to neutral pH, and vacuum dried at 90 °C for 24 h to, respectively, obtain BPC or NPC. Sodium tetraborate decahydrate (1 or 2 g) was also used to adjust the B content in the final codoped carbon material, and the final BPCs were denoted as BPC^1^ and BPC^2^, respectively. The PC catalyst was also prepared as above without the addition of secondary heteroatom precursors.

As references, BC and NC catalysts were prepared in another homogeneous system formed by dissolving cellulose (15 wt%) in zinc chloride aqueous solution (18.4 mol L^−1^). This was followed by the addition of a certain amount of sodium tetraborate decahydrate or dihydrodiamide and then carbonization at high temperatures (850 °C, 2 h) to prepare the single‐doped catalyst.

### Theoretical Calculations

All DFT calculations were completed using the Gaussian 16 program. The geometries of the involved species were optimized without any symmetry constraints. The B3LYP‐D3/6‐311G (d, p)‐(SMD, *n*‐hexane) level of theory was used unless otherwise noted. Subsequent frequency calculations (383 K) were employed to confirm the nature of the minimal point or **ts**. Calculations with larger basis sets (def2TZVPP) were further performed on the optimized structure to obtain a more precise Gibbs free energy. The relation of the **ts** to the reactants and products was determined using intrinsic reaction coordinates (IRCs). With the feed of the Gaussian checkpoint file, Mulliken charges, ELFs, TDOS, and PDOS were analyzed using Multiwfn 3.3.8 software.^[^ ^56^ ^]^

### Characterizations

The morphologies of the samples were studied by SEM (JEOL, JSM‐7500F) along with energy‐dispersive X‐ray spectroscopy and TEM (JEOL, JSM‐2100) using a TDY‐V5.2 image analysis system. Powder XRD was conducted in the 2*θ* range of 5°–70° with Cu K*α* radiation (PANalytical B.V., X'Pert Pro diffractometer). XPS was performed on a Thermo electron spectrometer (ThermoFisher, Thermo) to investigate the bonding characteristics. Brunauer–Emmett–Teller specific surface areas and pore size distributions were determined using nitrogen adsorption–desorption isotherms (Micromeritics Instrument, ASAP 2460). FTIR spectroscopy was conducted with a diamond attenuated total reflectance (ATR) attachment (Thermo Fisher, Nicolet Magna 560). Raman spectra were obtained in the back‐scattering mode using an Arion laser (532 nm) on a LabRAM Raman spectrometer (Horiba Jobin Yvon). Solid‐state ^31^P NMR measurements were collected on an Agilent 600 m with a magnetic field intensity of 14.1 T. The MAS rotating frequency was 10 kHz, and the chemical shift refers to H_3_PO_4_. The product yield analysis of the oxidation of alcohols was performed using gas chromatography–mass spectrometry (GC‐MS; Agilent 7890B, HP‐5 column) and quantitative analyses using gas chromatography (GC; Agilent 8860, HP‐5 column).

### Catalytic Oxidation of Reactants

The reactant (e.g., BA, 0.5 mmol) and 26 mg of catalyst were added to a 15 mL thick‐walled Teflon capped pressure glass tube with 4 mL of *n*‐hexane at 130 °C for 2 h. Oxygen (1 atm) was bubbled into the solution for 3 min before the reaction. After the reaction, the extracted liquid was removed through a syringe with a 0.22 µm organic filter followed by quantitative analyses using GC with dodecane as the internal standard.

## Conflict of Interest

The authors declare no conflict of interest.

## Author Contributions

H.Y. supervised the project. J.M. performed most of the experiments. Z.T. and H.S. participated in the experiments and DFT calculations. Y.L. and Q.X. participated in the results analysis and discussion. J.M. and H.Y. collectively analyzed all data and wrote the paper with supports from Q.P. and S.D. All authors contributed to the general discussion.

## Supporting information

Supporting Information

## Data Availability

The data that support the findings of this study are available in the Supporting Information of this article.
