# Supplementary material for: Metal‐Free Boron/Phosphorus Co‐Doped Nanoporous Carbon for Highly Efficient Benzyl Alcohol Oxidation
Source: Adv Sci (Weinh). 2022 Apr 11;9(17):2200518. doi: 10.1002/advs.202200518 (PMC9189657; doi:10.1002/advs.202200518)
Supplement: Supplementary file 1 — Supporting Information [file ADVS-9-2200518-s001.pdf]

ADVANCED SCIENCE
Open Access

Supporting Information

for *Adv. Sci.*, DOI 10.1002/advs.202200518

Metal-Free Boron/Phosphorus Co-Doped Nanoporous Carbon for Highly Efficient Benzyl Alcohol Oxidation

Juan Meng, Zhihan Tong, Haixin Sun, Yongzhuang Liu, Suqing Zeng, Jianing Xu, Qinqin Xia, Qingjiang Pan, Shuo Dou* and Haipeng Yu**

SUPPORTING INFORMATION

Metal-Free Boron/Phosphorus Co-Doped Nanoporous Carbon for Highly Efficient Benzyl Alcohol Oxidation

Juan Meng,[1] Zhihan Tong,[1] Haixin Sun,[1] Yongzhuang Liu,[1] Suqing Zeng,[1] Jianing Xu,[1] Qinqin Xia,[1] Qingjiang Pan,[2,*] Shuo Dou,[1,*] Haipeng Yu[1,*]

[1] Key Laboratory of Bio-based Material Science and Technology of Ministry of Education, Northeast Forestry University, Harbin 150040, China

[2] Key Laboratory of Functional Inorganic Material Chemistry, School of Chemistry and Materials Science, Heilongjiang University, Harbin 150080, China

[*] Corresponding: yuhaipeng20000@nefu.edu.cn; doushuo@nefu.edu.cn; panqjitc@163.com

This PDF file includes:

Scheme S1
Figures S1 – S22
Tables S1 – S8
References

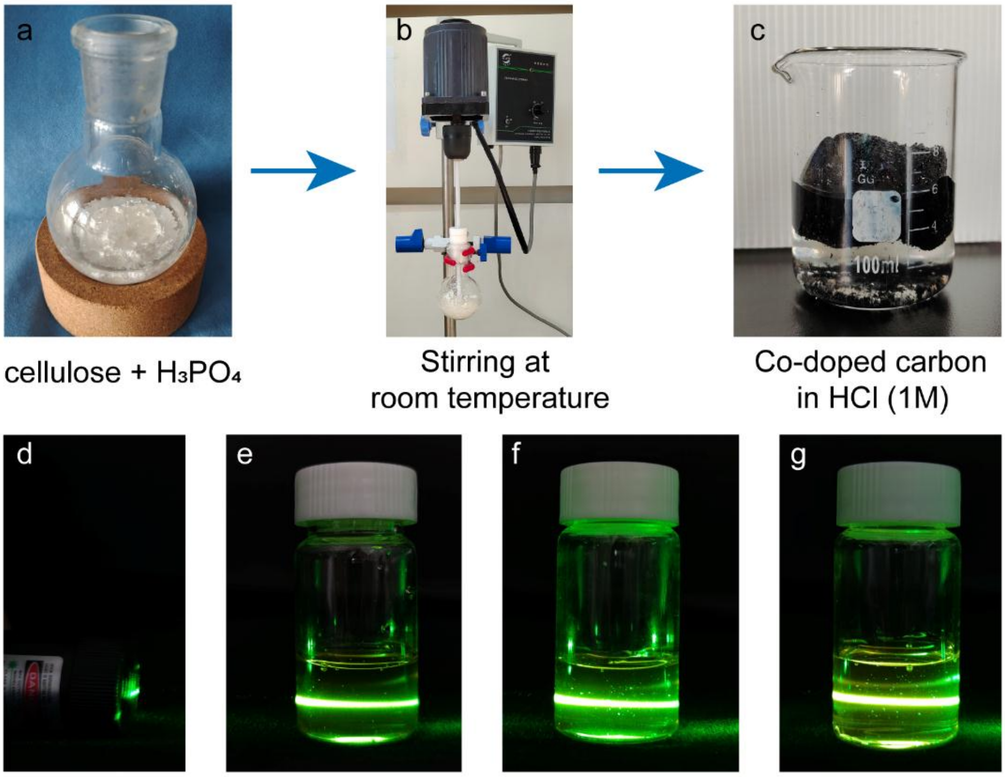

Scheme S1. Photograph illustration of preparing the B/N-PC catalysts. (a) Undissolved cellulose in phosphoric acid, (b) Stirring and dissolving the cellulose at room temperature, after it is completely dissolved, the second heteroatom source was added till completely dissolved, and (c) co-doped carbon materials were floating in 1M HCl. Photograph of supramolecular collosols in (d) laser pointer: (e) Cellulose-phosphoric acid supramolecular collosol, (f) cellulose/phosphoric acid-sodium tetraborate, decahydrate supramolecular collosol, and (g) cellulose-phosphoric acid-dicyandiamide supramolecular collosol.

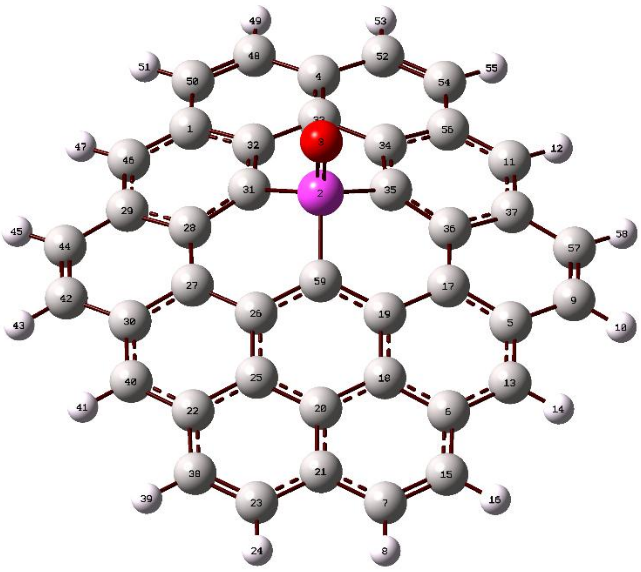

Fig. S1 Optimized geometric structure of PC.

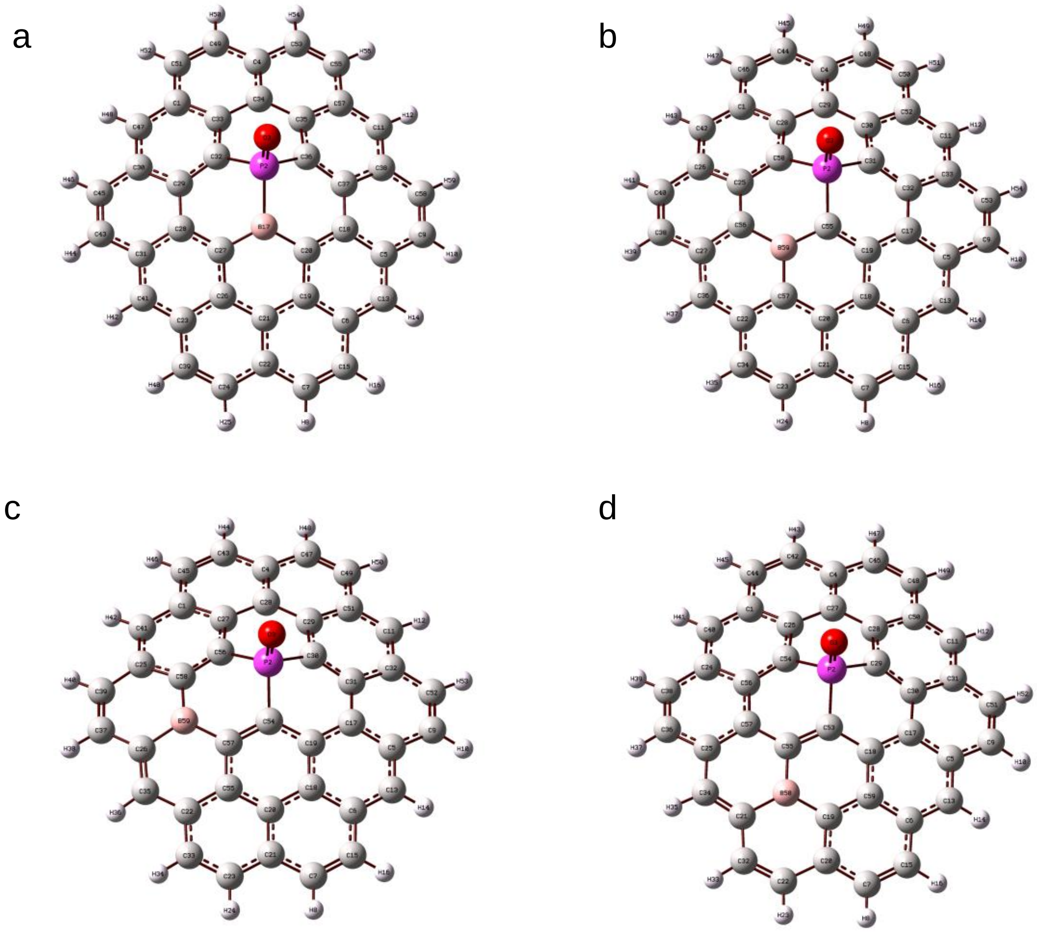

Fig. S2 Optimized geometric structures of (a) BPO, (b) BCPO, (c) $1BC_2PO$, (d) $2BC_2PO$ of BPC.

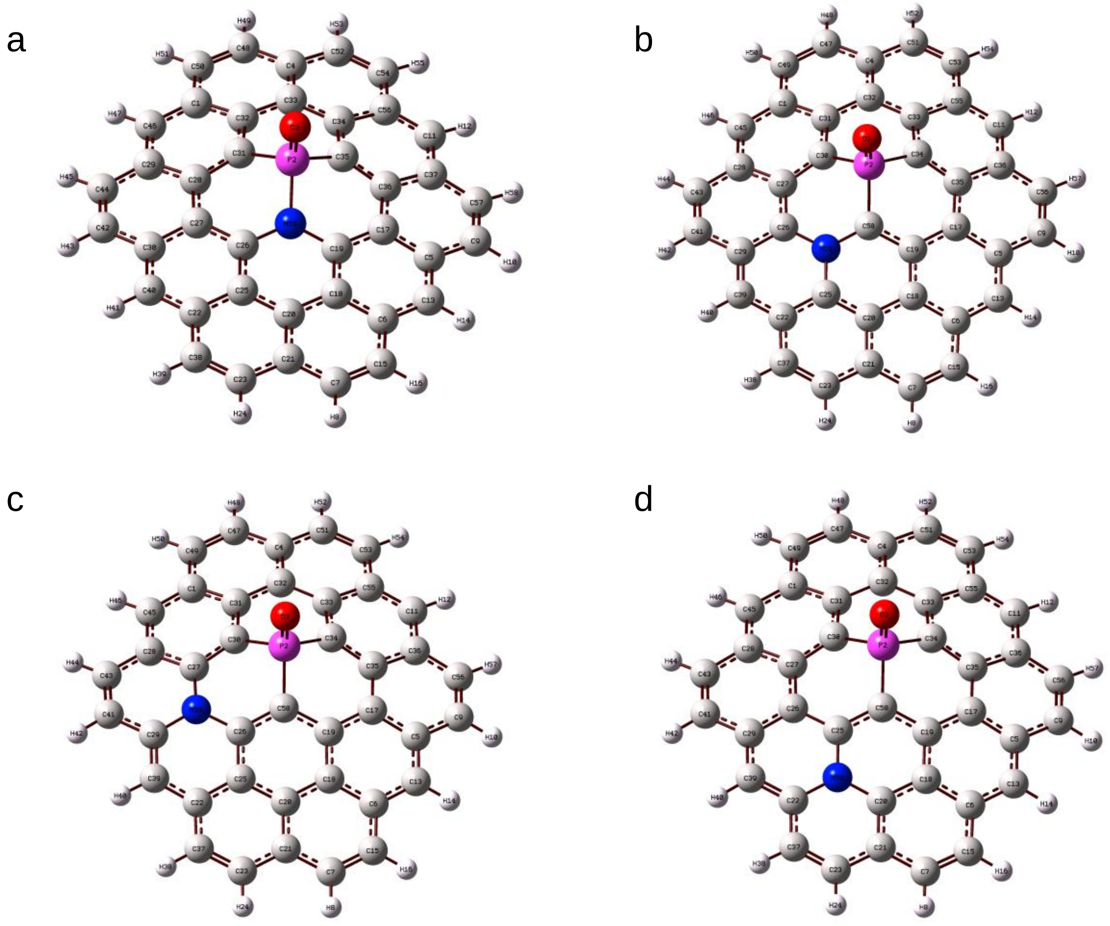

Fig. S3 Optimized geometric structures of (a) NPO, (b) NCPO, (c) $1NC_2PO$, (d) $2NC_2PO$ of NPC.

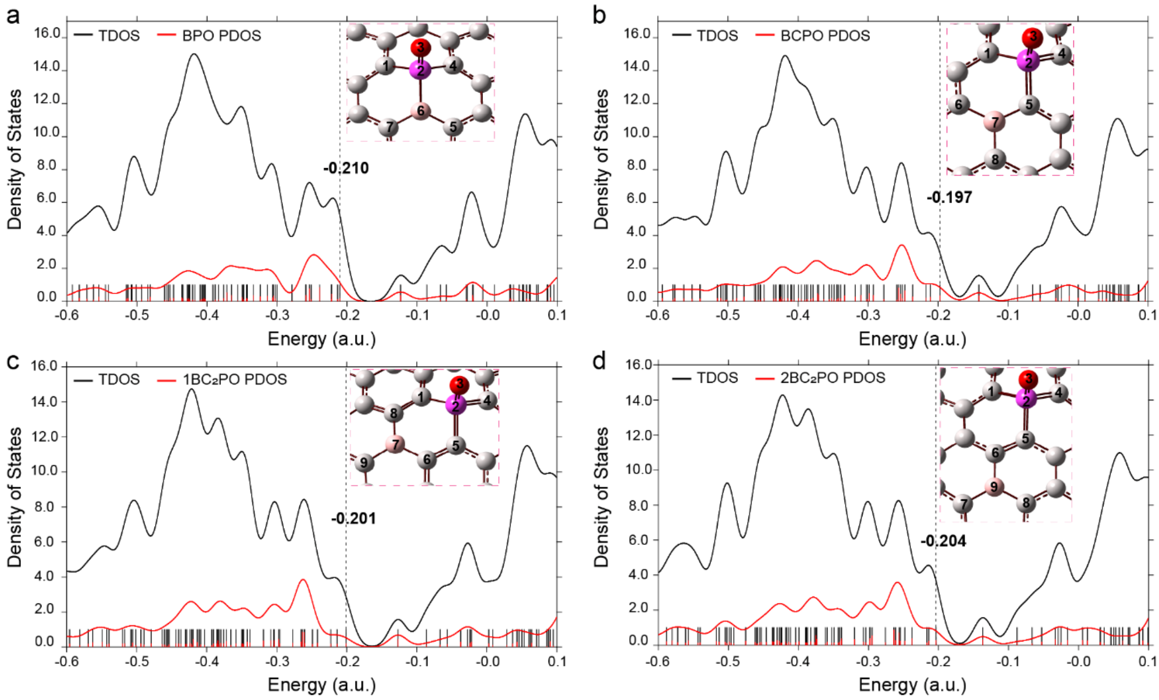

Fig. S4 TDOS and PDOS plots of different BPC configurations in several marked digital parts: (a) BPO, (b) BCPO, (c) 1BC_2PO, (d) 2BC_2PO, and the vertical dash lines correspond to HOMO levels at –0.210, –0.197, –0.201 and –0.204 a.u., respectively (insets are the configuration of different BPC moieties).

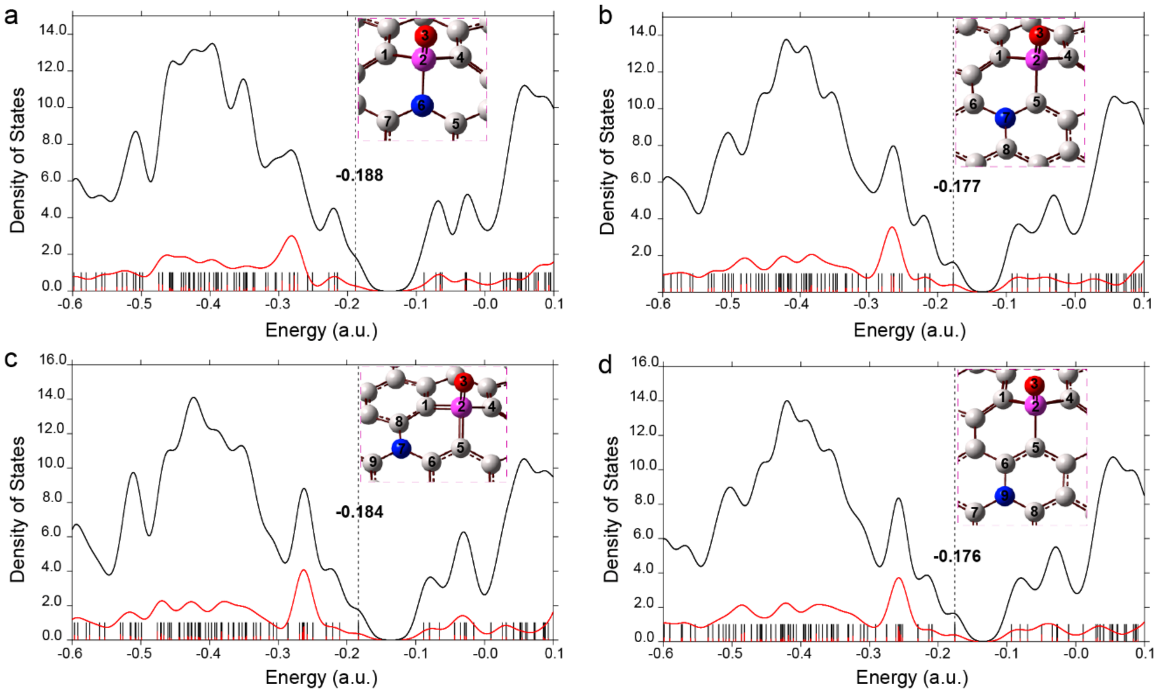

Fig. S5 TDOS and PDOS plots of different NPC configurations in several marked digital parts: (a) NPO, (b) NCPO, (c) $1NC_2PO$, (d) $2NC_2PO$, and the vertical dash lines correspond to HOMO levels at –0.188, –0.177, –0.184 and –0.176 a.u., respectively (insets are the configuration of different NPC moieties).

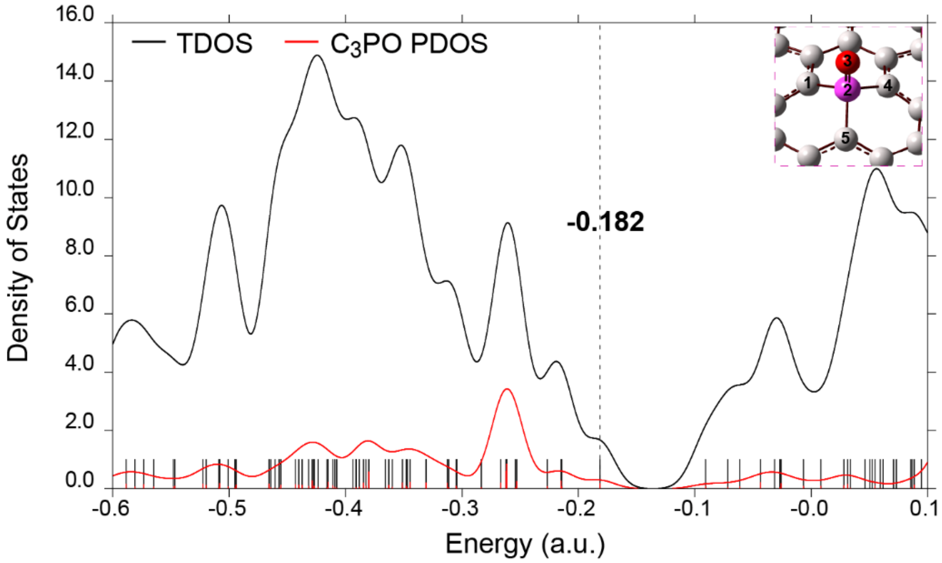

Fig. S6 Configuration and TDOS of PC with C_3PO and the vertical dash line corresponds to HOMO level at –0.182 a.u.

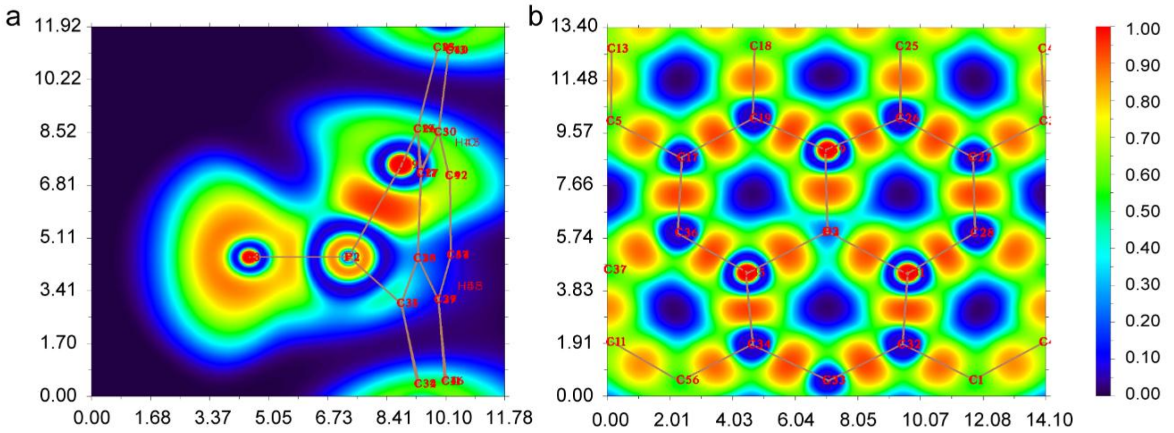

Fig. S7 Electron localization functions of C_3PO in PC (a) C-P=O on the side and (b) the top of graphite surface.

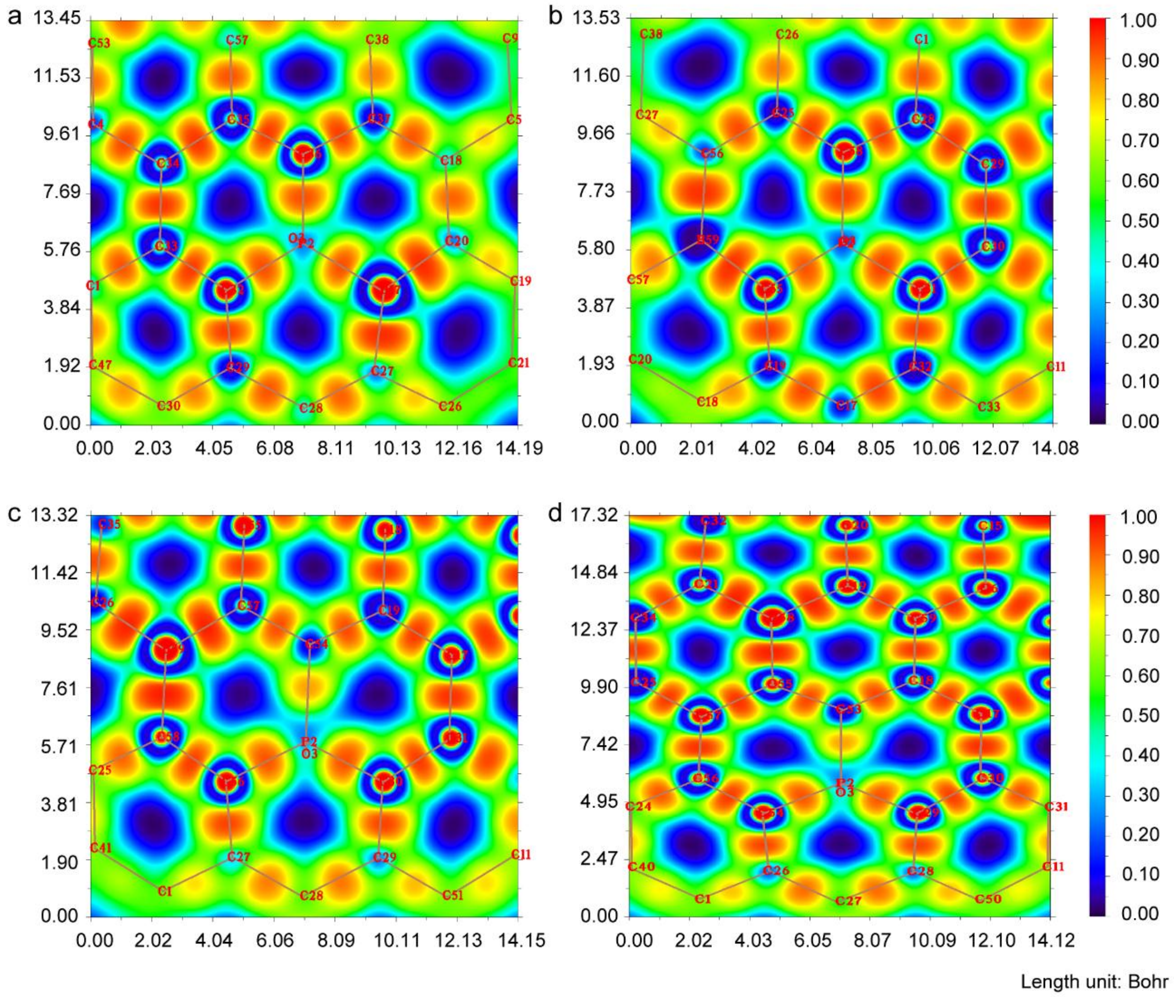

Fig. S8 Electron localization functions of the tops of graphite surfaces (a) BPO, (b) BCPO, (c) $1BC_2PO$, (d) $2BC_2PO$ of BPC.

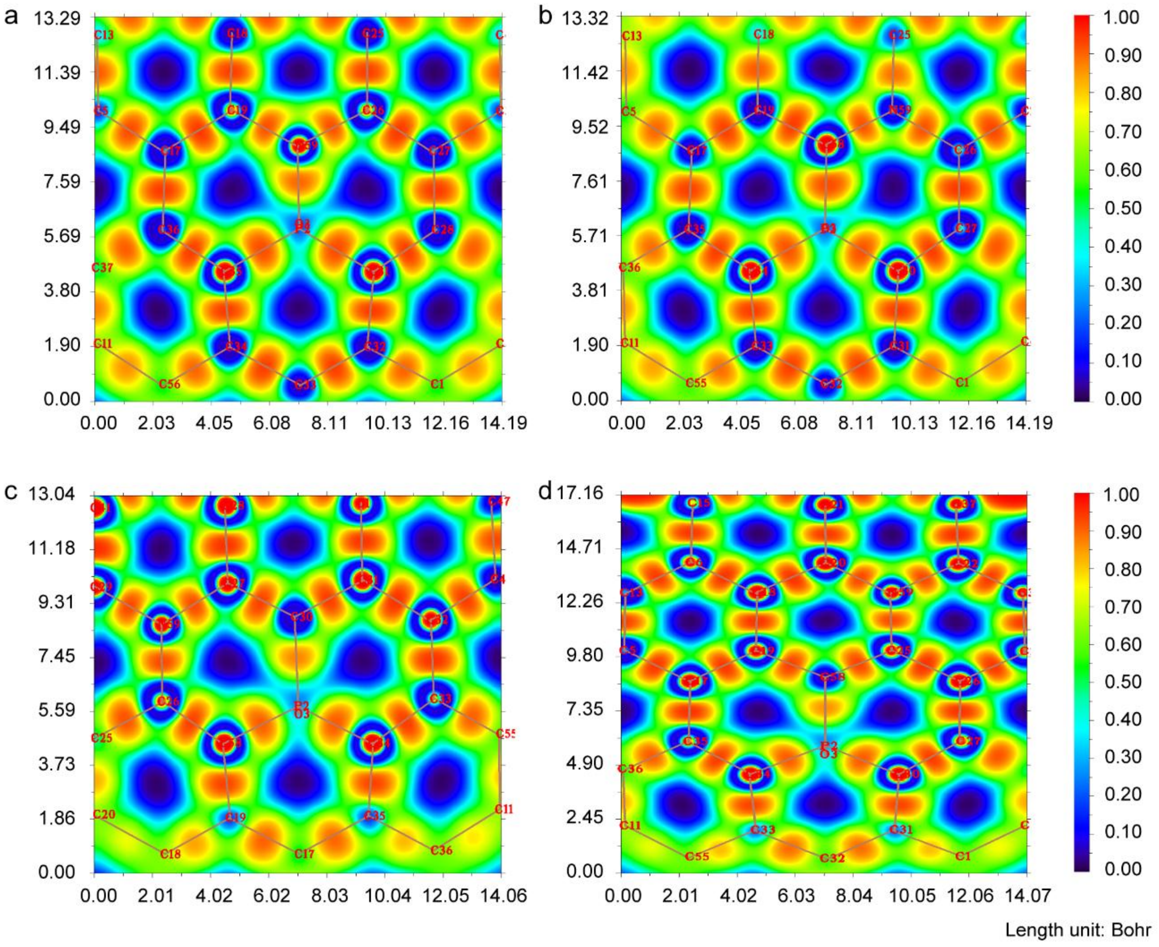

Fig. S9 Electron localization functions of the tops of graphite surfaces (a) NPO, (b) NCPO, (c) 1NC$_2$PO, (d) 2NC$_2$PO of NPC.

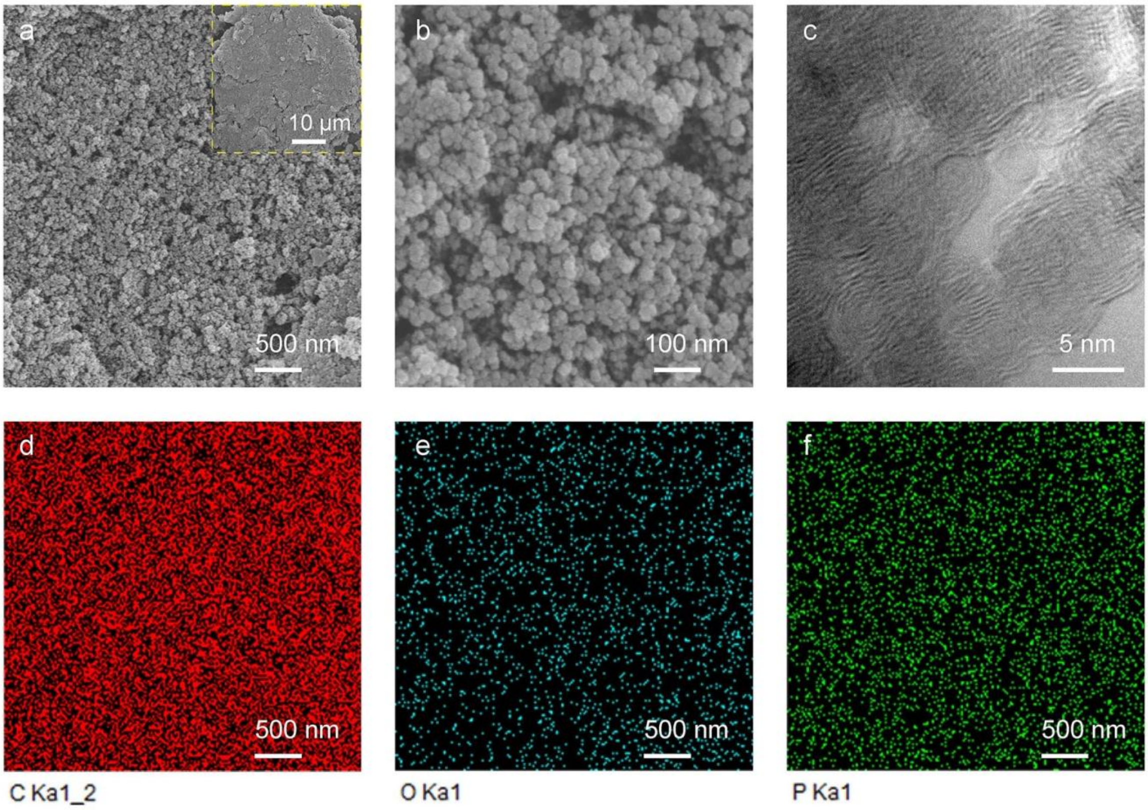

Fig. S10 Structure characterizations of PC. (a, b) SEM images. (c) HR-TEM image. (d-f) EDS mapping of C, O and P elements.

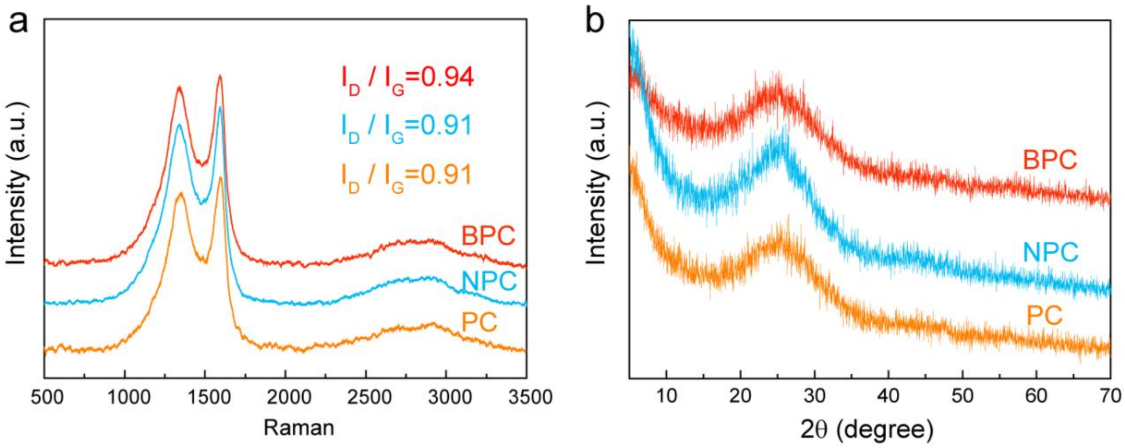

Fig. S11 (a) Raman spectra, and (b) XRD patterns of BPC, NPC and PC.

By comparing the Raman spectra of the three catalysts, the characteristic peaks of the D band at 1340 cm^{-1} and the G band at 1590 cm^{-1} are displayed in Figure S11a). The I_D/I_G value is above 0.9 for all the catalysts, and the BPC was slightly higher (0.94), indicating that all the doped carbon materials possessed much lattice defects, including disorder, edges and holes.[1] Moreover, the introduction of B appropriately increased the ratio of lattice defects in the carbon layer. These defects in carbon-based materials can provide more accessible sites for catalytic reactions, including better contact between the catalyst and the reactants in the solution to promote catalytic activity. XRD patterns were obtained to further study the crystal structure of the prepared carbon material (Figure S11b). The co-doped carbon materials all showed the typical diffraction pattern of graphitic carbon. Combining the above results, we can infer that the direct carbonization of the cellulose-phosphoric acid supramolecular collosol enables the high-level co-doping of nanoporous carbon, which may provide structural advantages for catalytic applications.

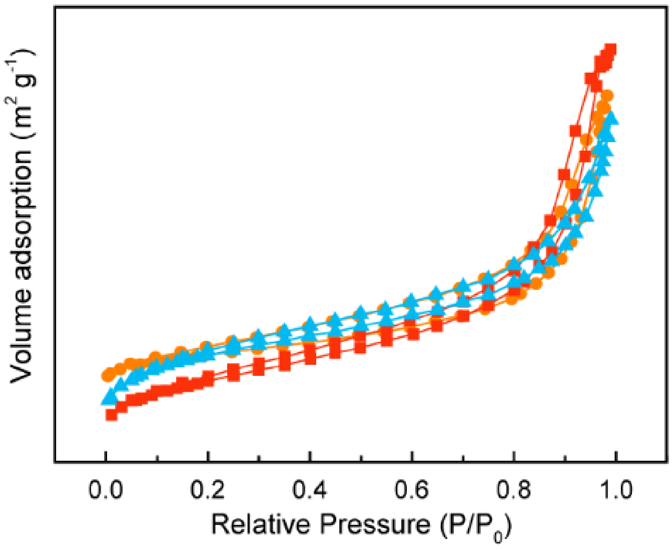

Fig. S12 Nitrogen adsorption-desorption isotherms of BPC, NPC, and PC.

The Brunauer-Emmett-Teller (BET) nitrogen adsorption-desorption measurement was performed to study the specific surface area and pore structure information of the doped carbon materials. All the samples showed type IV isotherms, indicating that the homogeneous carbonization of phosphoric acid and the introduction of other heteroatom source carbonization can still maintain a rich porous structure (Figure S12). BPC has the highest specific surface area (1526 m^2 g^{-1}) and total pore volume, which was much higher than that of others-doped carbon (Table S3). The introduction of the second heteroatom has influence on the specific surface area of carbon materials. Therefore, both BPC and NPC with high surface area, nanoporous structure and large pore volume can provide channels for the transportation and diffusion of reaction materials and promote catalytic kinetics.[2]

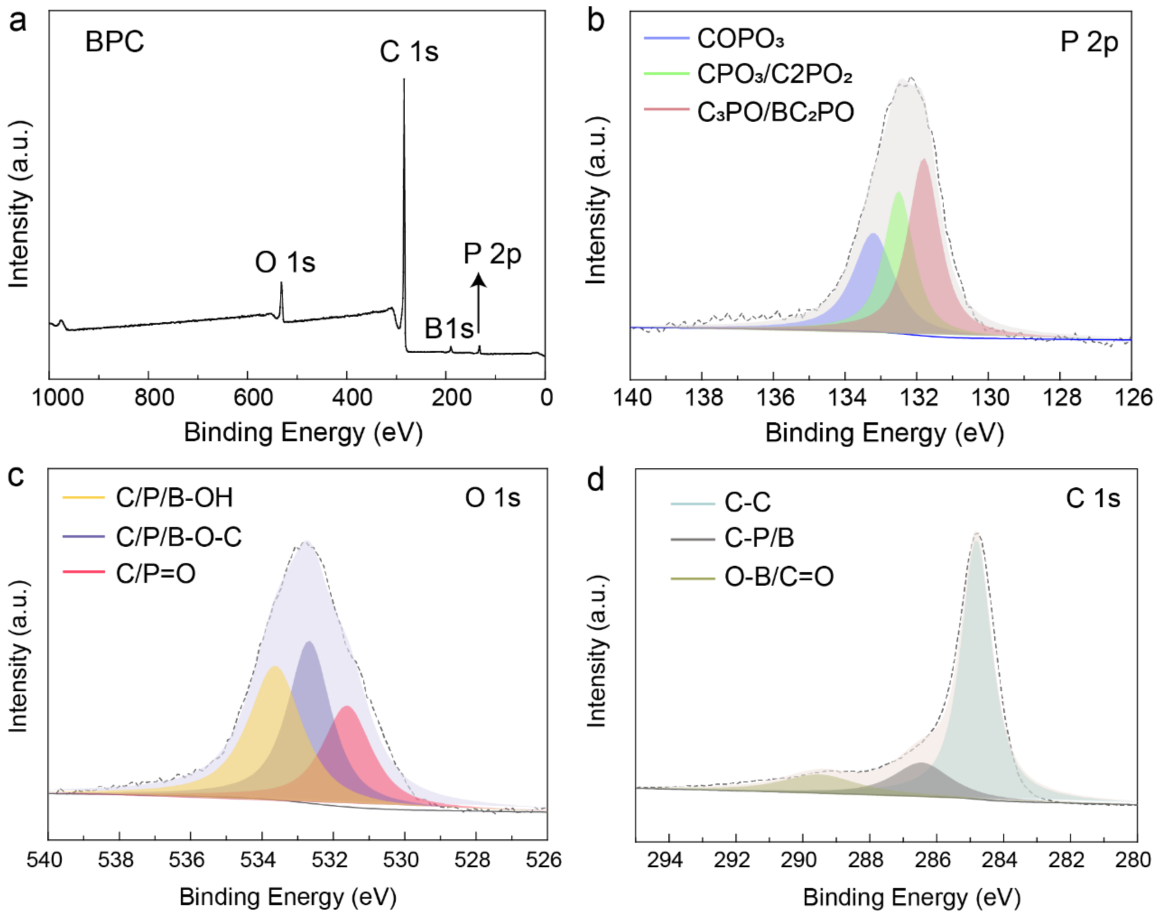

Fig. S13 (a) XPS survey spectra, and the deconvolution peaks of P 2p (b), O 1s (c) and C1s (d) of BPC.

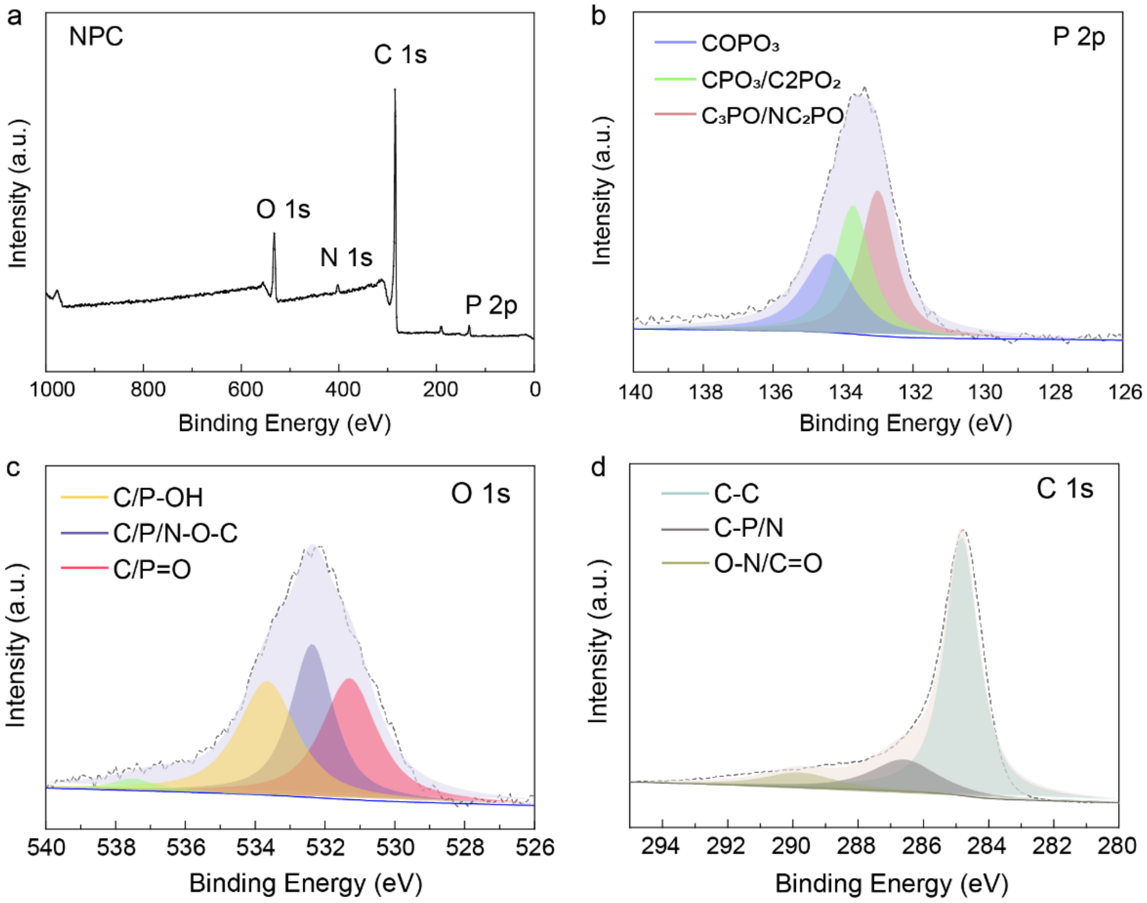

Fig. S14 (a) XPS survey spectra, and the deconvolution peaks of P 2p (b), O 1s (c) and C1s (d) of NPC.

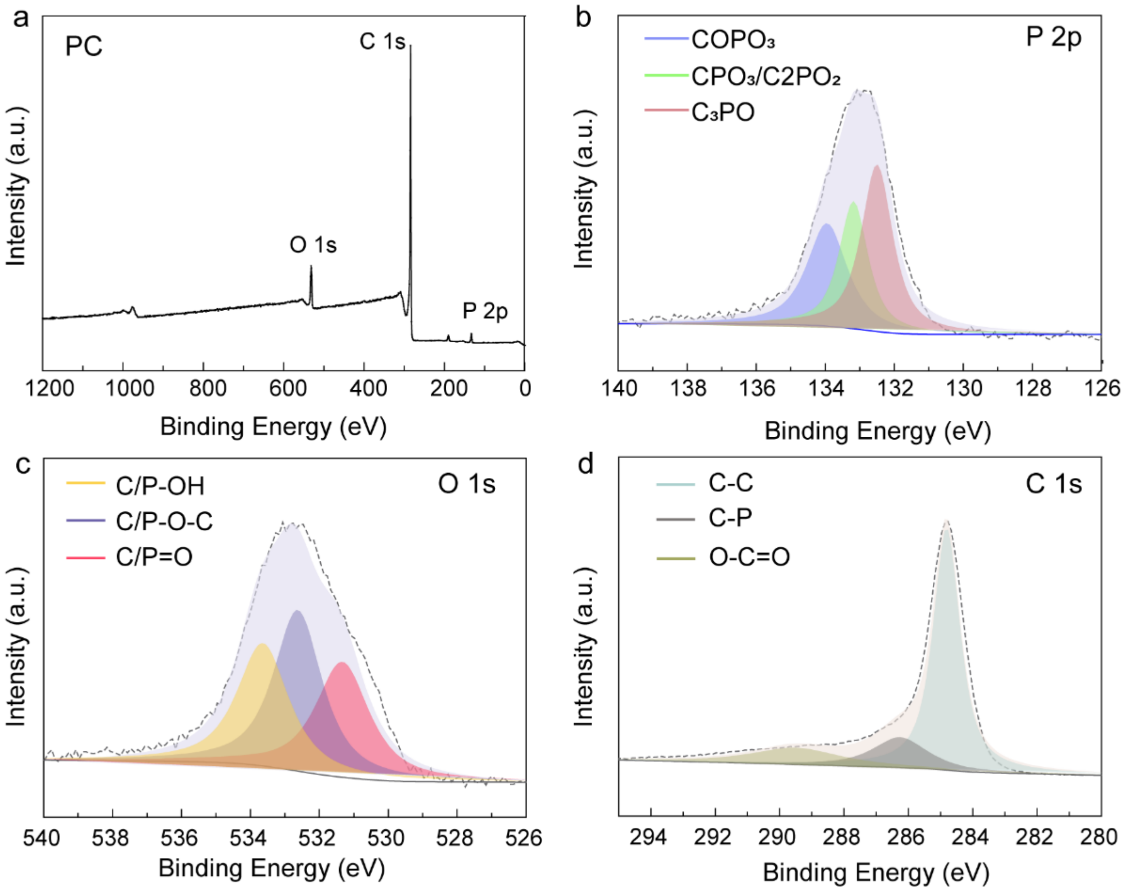

Fig. S15 (a) XPS survey spectra, and the deconvolution peaks of P 2p (b), O 1s (c) and C1s (d) of PC.

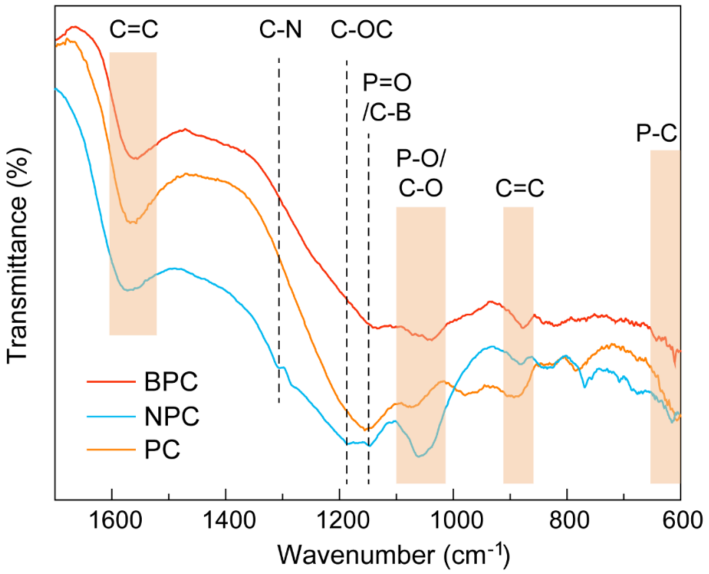

Fig. S16 FTIR spectra of BPC, NPC and PC.

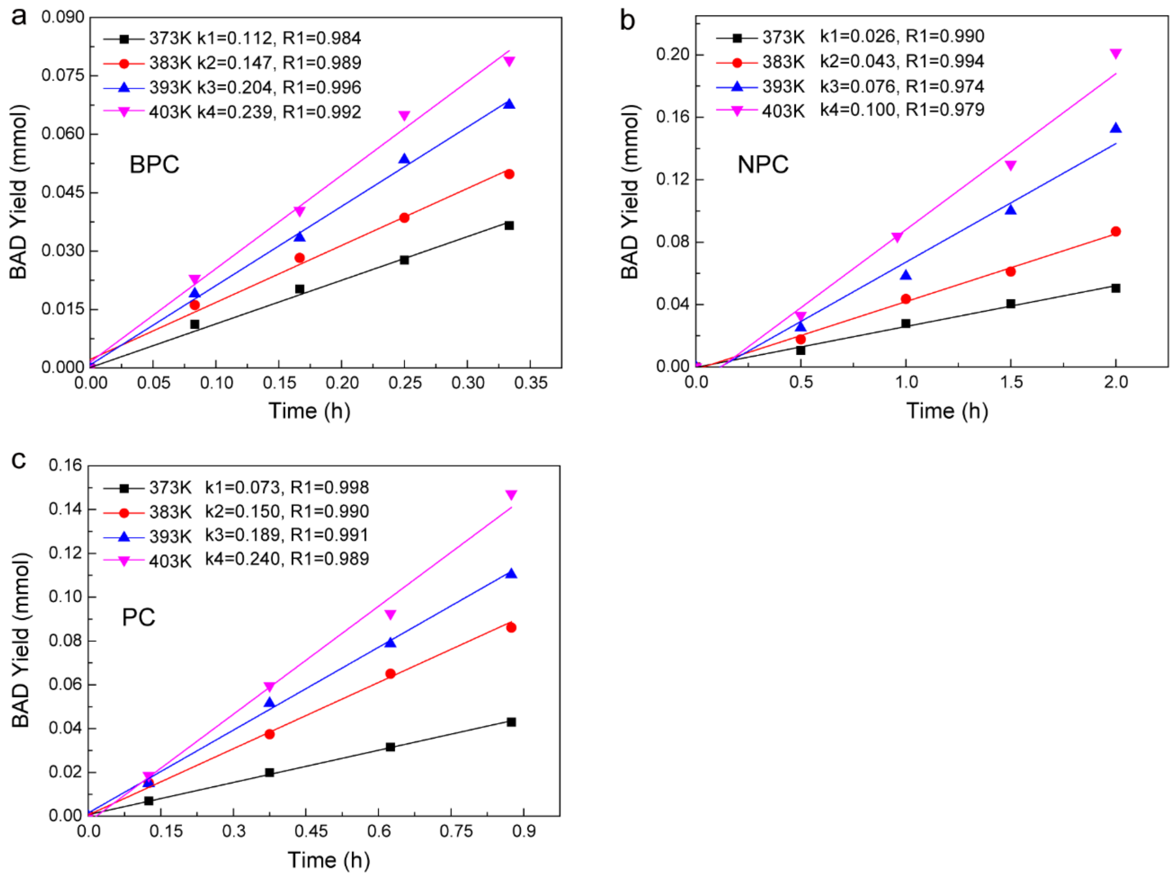

Fig. S17 Effects of the rate constant (*k*) of oxidation of benzyl alcohol. (a) BPC, (b) NPC and (c) PC at different temperatures. Reaction conditions: 0.5 mmol benzyl alcohol, 26 mg catalyst, 4 mL *n*-hexane, and 1 atm O_2.

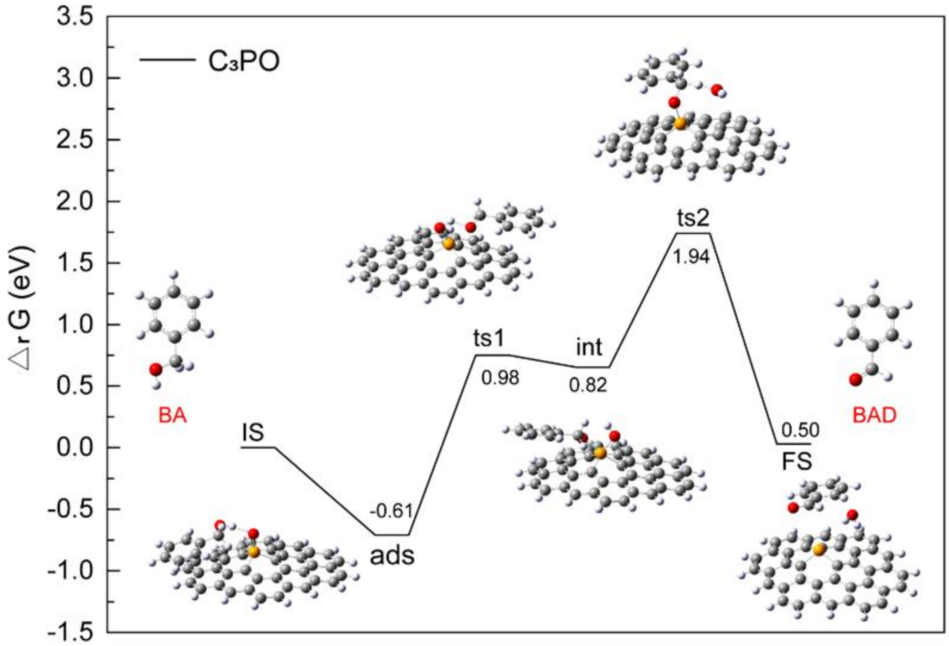

Fig. S18 DFT studies on the catalytic reaction mechanism of C_3PO configurations of PC for BA oxidation. Required energies of adsorption (**ads**), intermediate formation (**int**), and transition state formation (**ts**) during the BA stepwise oxidation. The C, O, P and H atoms are given in gray, red, yellow and white colors, respectively.

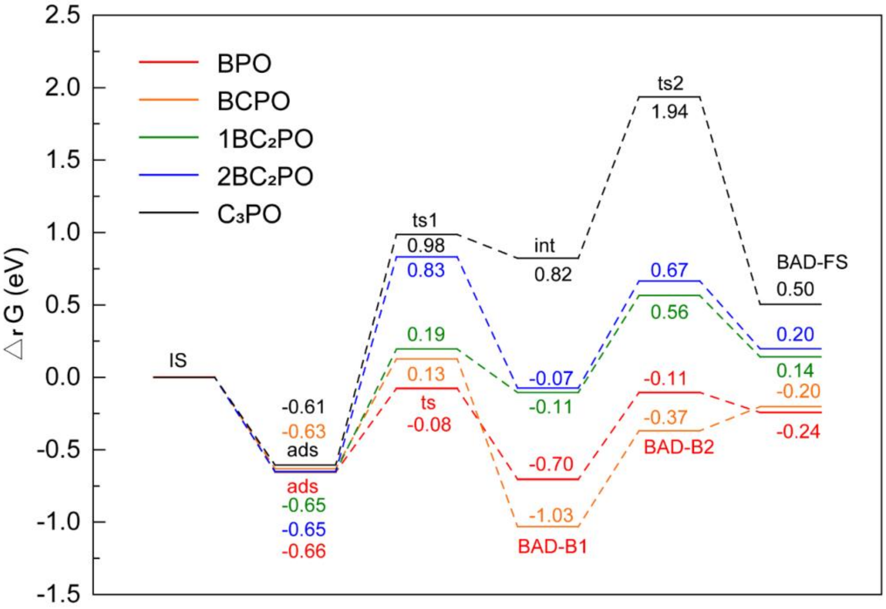

Fig. S19 DFT studies on the catalytic reaction mechanism of BPO, BCPO, $1BC_2PO$ and $2BC_2PO$ configurations of BPC, and C_3PO of PC for BA oxidation, respectively. Marked energies for the target product BAD adsorbed on the intermediate structure of the BPO (**BAD-B1**), BAD desorption and intermediate state of BPO (**BAD-B2**), adsorption (**ads**), intermediate formation (**int**), and transition state formation (**ts**) during the stepwise oxidation of the models.

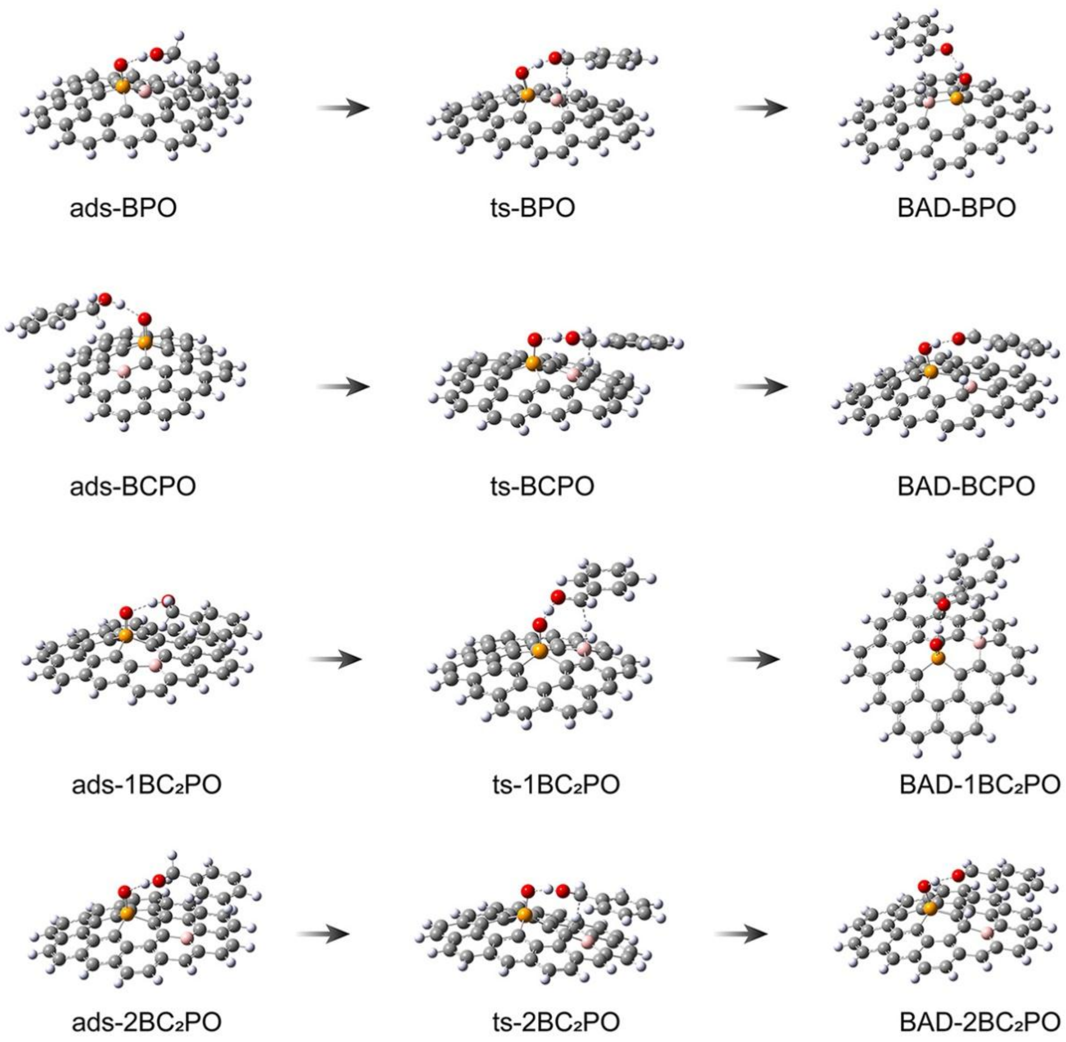

Fig. S20 The structures of adsorption (**ads**), intermediate formation (**int**), and transition state formation (**ts**) for the target product BAD (**BAD-BPC**) during the stepwise oxidation of the models. The C, O, P, B and H atoms given in gray, red, yellow, pink and white color, respectively.

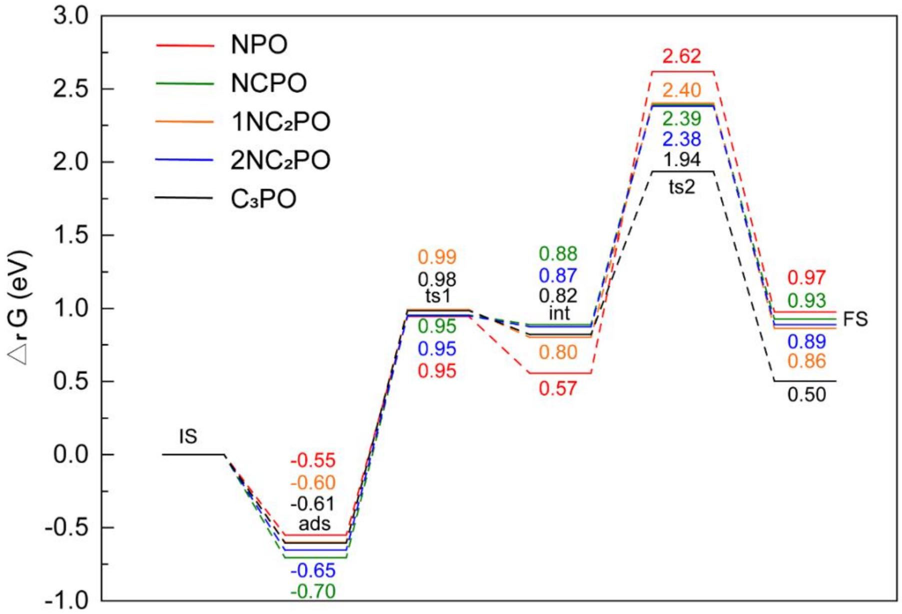

Fig. S21 DFT studies on the catalytic reaction mechanism of NPO, NCPO, $1NC_2PO$ and $2NC_2PO$ configurations of NPC and C_3PO of PC for BA oxidation, respectively. Required energies of **ads**, **int**, and **ts** during the BA stepwise oxidation.

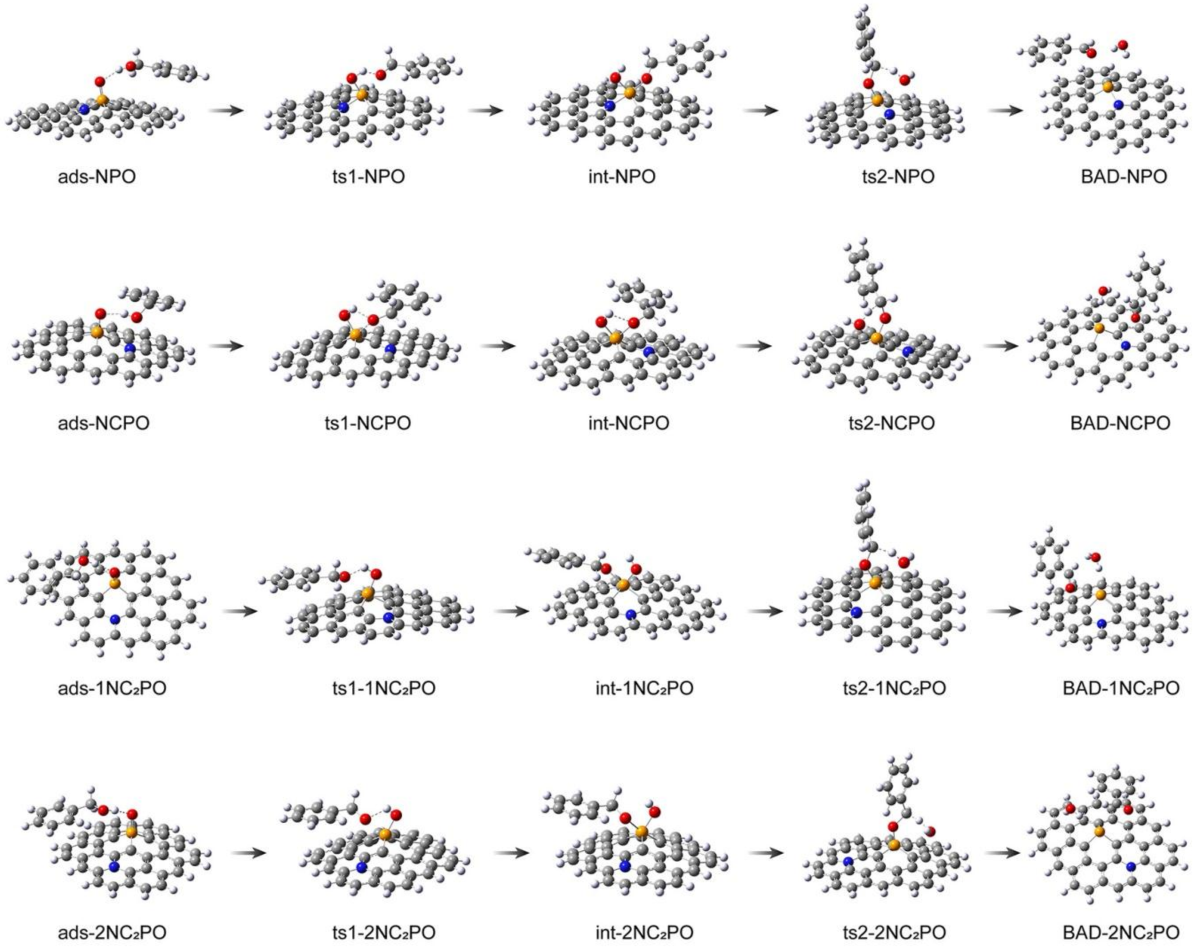

Fig. S22 The structures of adsorption (**ads**), intermediate formation (**int**), and transition state formation (**ts**) for the target product BAD (BAD-NPC) during the stepwise oxidation of the models. The C, O, P, N and H atoms given in gray, red, yellow, blue and white, respectively.

Table S1. Mulliken charges analysis of the active sites in PC and different BPC configurations (the atomic numbers are the same as that in Fig. S1).

**C_3PO /e**		**BPO /e**		**BCPO /e**		**1BC_2PO /e**		**2BC_2PO /e**	
**O3**	-0.558	**O3**	-0.561	**O3**	-0.565	**O3**	-0.559	**O3**	-0.556

**P2**	1.185	**P2**	0.882	**P2**	1.095	**P2**	1.107	**P2**	1.179
**C31**	-0.402	**C32**	-0.391	**C58**	-0.388	**C56**	-0.416	**C54**	-0.398
**C35**	-0.402	**C36**	-0.391	**C31**	-0.399	**C30**	-0.427	**C29**	-0.400
**C59**	-0.401	**B17**	0.508	**C55**	-0.593	**C54**	-0.425	**C53**	-0.374
**C26**	-0.105	**C20**	-0.216	**B59**	0.748	**C57**	-0.131	**C55**	-0.211
**C27**	0.163	**C27**	-0.216	**C56**	-0.175	**B59**	0.769	**B58**	0.770
				**C57**	-0.205	**C58**	-0.170	**C19**	-0.249
						**C26**	-0.338	**C21**	-0.323

When B directly covalently bonded to P, the positive charge of both the heteroatom B (+0.508 e) and P (+0.882 e) were the lowest due to their lower electronegativity, and when the intermediate contains the more electronegative C atom, the positive charges of both B and P increased significantly, indicating the secondary regulation of the charge of the C atom in graphite structure.

Table S2. Mulliken charges analysis of the active sites part in PC and different NPC configurations (the atomic numbers are the same as that in Fig. S2).

C_3PO /e		NPO /e		NCPO /e		$1NC_2PO$ /e		$2NC_2PO$ /e	
**O3**	-0.558	**O3**	-0.533	**O3**	-0.549	**O3**	-0.556	**O3**	-0.560
**P2**	1.185	**P2**	1.175	**P2**	1.119	**P2**	1.161	**P2**	1.167
**C31**	-0.402	**C31**	-0.401	**C30**	-0.354	**C30**	-0.396	**C30**	-0.394
**C35**	-0.402	**C35**	-0.401	**C34**	-0.397	**C34**	-0.394	**C34**	-0.395
**C59**	-0.401	**N59**	-0.908	**C58**	-0.053	**C58**	-0.397	**C58**	-0.550
**C26**	-0.105	**C19**	0.419	**N59**	-0.645	**C26**	0.408	**C25**	0.487
**C27**	0.163	**C26**	0.420	**C25**	0.396	**N59**	-0.723	**N59**	-0.698
				**C26**	0.264	**C27**	0.363	**C20**	0.364
						**C29**	0.333	**C22**	0.327

Table S3. Textural properties of the PC as well as the co-doped carbon catalysts.

**Sample name**	$\boldsymbol{S_{BET}}$ **(m^2g^{-1})**	$\boldsymbol{V_{total}}$ **(cm^3g^{-1})**	$\boldsymbol{D_v}$**(d) (nm)**
BPC	1526	1.57	3.06
NPC	1041	2.22	2.21
PC	1349	1.29	3.82

S_{BET} means the specific surface area; V_{total} is the total pore volume; D_v(d) is the pore diameter of BJH desorption.

Table S4. Total and calculated element contents in BPC, NPC, PC, BC, and NC catalysts.

Catalyst	C (at. %)	O (at. %)	P (at. %)	B (at. %)	N (at. %)
BPC	85.94	8.54	2.35	3.17	--
BPC[1]	87.66	8.19	2.10	2.05	
BPC[2]	85.10	9.43	1.70	3.77	
NPC	88.53	5.22	2.18	--	4.07
PC	89.30	8.43	2.27	--	--
BC	90.44	6.81	--	2.76	--
NC	86.24	9.72	--	--	4.04

Table S5. The comparison of catalytic activity with different catalysts for BA oxidation.

Substrate (mmol)	Oxidant	Catalyst (mg)	Temp. (˚C)	Time (h)	Yield (%)	Selectivity (%)	TOF[b] (mol·g^{-1} h^{-1}×10^{-3})	Ref.
**0.5**	**1 atm O_2**	**BPC/26**	**130**	**2**	**91.4**	**95.1**	**8.8**	This work
0.5	1 atm O_2	PC/26	130	2	54.6	84.8	5.2	
0.5	1 atm O_2	NPC/26	130	24	19.4	69.6	1.9	
1.9	air	50wt%GO	150	24	27.0	>99	0.67	[3]
0.25	2 atm O_2	modified GO/50	100	30	93.1	>99	0.16	[4]
0.1	1 atm O_2	NG/30	80	10	12.8	>98	0.05	[5]
1.1	air	N-AC/100	120	5	10.0	>99	0.51	[6]
0.33	1 atm O_2	CTF-1-400/8.5[c]	100	0.75	61.0	>99	--	[7]
1.0	1 atm O_2	P-doped porous carbon/50	100	24	56.1	95.7	0.48	[8]
0.5	1 atm O_2	P-doped carbon/50	100	24	79.8	>99	0.33	[9]
100μL	1 atm O_2	PS-Gc/50	100	24	67.3	91.0	0.38	[10]
0.5	0.5atm O_2	G1000 (S and O co-dopant)/50	120	5	95.0	96.7	1.90	[11]
0.2	H_2O_2/125 μL	Co/P-NC/26	100	20	89.1	99.0	--	[12]

[b] Turnover frequency (TOF) = $\frac{\text{mole of BAD}}{\text{mass of catalyst (g)} \times \text{reaction time (h)}}$

[c] In the presence of Cs_2CO_3 as the base.

Table S6. BA Oxidation Catalyzed by various MPC carbon catalysts in n-hexane.[a]

Catalyst	BAD Yield (%)	BAD Selectivity (%)	TOF[b] ($\times 10^{-3}$)
**BPC**	**91.4**	**95.1**	**8.8**
BPC[1]	73.8	93.8	7.1
BPC[2]	83.4	91.9	8.0
NPC	19.4	69.6	1.9
PC	54.6	84.8	5.2
BC	11.8	84.4	1.1
NC	10.6	80.9	1.0
C	4.6	81.2	0.5

[a] Reaction conditions: 26 mg catalyst, 0.5 mmol substrate, 4ml *n*-hexane, 1 atm O_2, 130 ^{o}C, 2 hours.

[b] Turnover frequency (TOF) = $\frac{\text{mole of BAD}}{\text{mass of catalyst (g)} \times \text{reaction time (h)}}$

Table S7. The reaction energies (eV) during stepwise BA oxidation in BPC and PC.

**BPC**	**ads**	**ts**	**BAD-B1**	**BAD-B2**	**FS**
**BPO**	-0.66	-0.08	-0.7	-0.11	-0.24
**BCPO**	-0.63	0.13	-1.03	-0.37	-0.20
**1BC_2PO**	-0.65	0.19	-0.11	0.56	0.14
**2BC_2PO**	-0.65	0.83	-0.07	0.67	0.20
**C_3PO-PC**	-0.61	0.98	0.82	1.94	0.50

Table S8. The reaction energies (eV) during stepwise BA oxidation in NPC and PC.

**NPC**	**ads**	**ts1**	**int**	**ts2**	**FS**
**NPO**	-0.55	0.95	0.57	2.62	0.97
**NCPO**	-0.70	0.95	0.88	2.39	0.93
**1NC_2PO**	-0.60	0.99	0.80	2.40	0.86
**2NC_2PO**	-0.65	0.95	0.87	2.38	0.89
**C_3PO-PC**	-0.61	0.98	0.82	1.94	0.50

References

[1] W. Li, D. Wang, Y. Zhang, L. Tao, T. Wang, Y. Zou, Y. Wang, R. Chen and S. Wang, *Advanced Materials* **2020**, *32*, 1907879.

[2] S. Dou, X. Wang and S. Wang, *Small Methods* **2019**, *3*, 1800211.

[3] D. R. Dreyer, H. P. Jia and C. W. Bielawski, *Angewandte Chemie* **2010**, *122*, 6965-6968.

[4] S. Zhu, Y. Cen, M. Yang, J. Guo, C. Chen, J. Wang and W. Fan, *Applied Catalysis B: Environmental* **2017**, *211*, 89-97.

[5] J. Long, X. Xie, J. Xu, Q. Gu, L. Chen and X. Wang, *ACS Catalysis* **2012**, *2*, 622-631.

[6] H. Watanabe, S. Asano, S.-i. Fujita, H. Yoshida and M. Arai, *ACS Catalysis* **2015**, *5*, 2886-2894.

[7] S. Abednatanzi, P. G. Derakhshandeh, K. Leus, H. Vrielinck, F. Callens, J. Schmidt, A. Savateev and P. Van Der Voort, *Science Advances* **2020**, *6*, eaaz2310.

[8] M. A. Patel, F. Luo, M. R. Khoshi, E. Rabie, Q. Zhang, C. R. Flach, R. Mendelsohn, E. Garfunkel, M. Szostak and H. He, *ACS Nano* **2016**, *10*, 2305-2315.

[9] X. Hu, M. Fan, Y. Zhu, Q. Zhu, Q. Song and Z. Dong, *Green Chemistry* **2019**, *21*, 5274-5283.

[10] M. A. Patel, F. Luo, K. Savaram, P. Kucheryavy, Q. Xie, C. Flach, R. Mendelsohn, E. Garfunkel, J. V. Lockard and H. He, *Carbon* **2017**, *114*, 383-392.

[11] S. Zhu, Y. Chen, X. Gao, Z. Lv, Y. He, J. Wang and W. Fan, *Catalysis Science & Technology* **2020**, *10*, 2786-2796.

[12] S. Ji, Y. Chen, Z. Zhang, W.-C. Cheong, Z. Liu, D. Wang and Y. Li, *Nanoscale Horizons* **2019**, *4*, 902-906.
